# Influence of common reference regions on regional tau patterns in cross-sectional and longitudinal [^18^F]-AV-1451 PET data

**DOI:** 10.1016/j.neuroimage.2021.118553

**Published:** 2021-09-03

**Authors:** Christina B. Young, Susan M. Landau, Theresa M. Harrison, Kathleen L. Poston, Elizabeth C. Mormino, ADNI

**Affiliations:** a Department of Neurology and Neurological Sciences, Stanford University School of Medicine, Stanford, CA United States; b Helen Wills Neuroscience Institute, University of California Berkeley, Berkeley, CA United States

**Keywords:** AV-1451, Flortaucipir, Tau pet, Longitudinal tau pet, Reference region

## Abstract

Tau PET has allowed for critical insights into *in vivo* patterns of tau accumulation and change in individuals early in the Alzheimer’s disease (AD) continuum. A key methodological step in tau PET analyses is the selection of a reference region, but there is not yet consensus on the optimal region especially for longitudinal tau PET analyses. This study examines how reference region selection influences results related to disease stage at baseline and over time. Longitudinal flortaucipir ([18F]-AV1451) PET scans were examined using several common reference regions (*e.g*., eroded subcortical white matter, inferior cerebellar gray matter) in 62 clinically unimpaired amyloid negative (CU A-) individuals, 73 CU amyloid positive (CU *A*+) individuals, and 64 amyloid positive individuals with mild cognitive impairment (MCI *A*+) from the Alzheimer’s Disease Neuroimaging Initiative (ADNI). Cross-sectionally, both reference regions resulted in robust group differences between CU A-, CU *A*+, and MCI *A*+ groups, along with significant associations with CSF phosphorylated tau (pTau-181). However, these results were more focally specific and akin to Braak Staging when using eroded white matter, whereas effects with inferior cerebellum were globally distributed across most cortical regions. Longitudinally, utilization of eroded white matter revealed significant accumulation greater than zero across more regions whereas change over time was diminished using inferior cerebellum. Interestingly, the inferior temporal target region seemed most robust to reference region selection with expected cross-sectional and longitudinal signal across both reference regions. With few exceptions, baseline tau did not significantly predict longitudinal change in tau in the same region regardless of reference region. In summary, reference region selection deserves further evaluation as this methodological step may lead to disparate findings. Inferior cerebellar gray matter may be more sensitive to cross-sectional flortaucipir differences, whereas eroded subcortical white matter may be more sensitive for longitudinal analyses examining regional patterns of change.

## 1. Introduction

Beta-amyloid (A*β*) plaques and tau neurofibrillary tangles are present years before a clinical diagnosis of Alzheimer’s disease (AD) (Villemagne et al., 2013; Hanseeuw et al., 2019). Although initial longitudinal tau PET studies have shed insight on the patterns of tau change over time, the impact of methodological steps such as reference region selection in these analyses remains unclear. Cross-sectional studies commonly use inferior cerebellar gray matter (GM) or whole cerebellum as the reference region given minimal neurofibrillary tangle pathology in this region (Baker et al., 2017). In contrast, longitudinal tau PET studies vary in their reference region selection and often use either inferior cerebellar GM (Jack et al., 2018; Knopman et al., 2020; Franzmeier et al., 2020; Vogel et al., 2020; Chiotis et al., 2018; Sintini et al., 2020; Utianski et al., 2020; Sintini et al., 2019) or subcortical white matter (WM) (Hanseeuw et al., 2019; Harrison et al., 2019). This divergence between cross-sectional and longitudinal reference region selection parallels work investigating longitudinal amyloid PET with florbetapir, which has shown that a large composite region consisting of subcortical WM along with cerebellum may provide a more stable estimates of change over time (Landau et al., 2015). These methodological decisions are particularly important for the integration of tau PET into clinical trials, which has become commonplace in multiple high profile anti-amyloid trials investigating aducanamab (Sevigny et al., 2016), solanezumab (Siemers et al., 2016), lecanemab (Swanson et al., 2021), and donanamab (Mintun et al., 2021). In the context of these clinical trials, tau PET represents a key modality that can provide insight into disease modification answering questions such as whether removal of amyloid results in downstream changes to tau PET signal (Cummings and Fox, 2017). In these large-scale studies it is not possible to perform gold-standard dynamic imaging along with arterial sampling (Barret et al., 2017; Wooten et al., 2017; Hahn et al., 2017) to enable true quantification of the PET signal. Thus, there is an urgent need to evaluate processing pipelines that can be applied in the context of large multi-site clinical trials. It is also likely that the interpretation of tau PET signal in clinical trials of AD will be more complex than amyloid PET, given the focal nature of tau PET signal (Schwarz et al., 2016) along with greater sources of off-target binding in tau PET ligands (Leuzy et al., 2019; Lemoine et al., 2018).

Given that tau PET data is being increasingly integrated, but there is a lack of knowledge regarding the impact of fundamental processing decisions regarding simplified analyses, we sought to directly compare the pattern of results related to disease stage across common reference regions. Post-mortem studies have highlighted initial tau deposition within entorhinal cortex (EC) and medial temporal lobe, followed by spread into neocortex beginning in the inferior temporal (IT) cortex (Braak and Braak, 1991) that is linked with abnormal amyloid levels (Nelson et al., 2012). Cross-sectional tau PET studies examining *in vivo* tau deposition in clinically unimpaired (CU) individuals, those with mild cognitive impairment (MCI), and those with AD have largely confirmed these postmortem observations and have provided additional insights regarding patterns in cortex. In addition to IT, lateral inferior parietal (IP) and rostral middle frontal gyrus (rMFG) have been noted in CU amyloid positive (A+) individuals that transition to MCI (Betthauser et al., 2020) and in comparisons between clinically impaired versus CU individuals using tau PET (Lowe et al., 2018). These studies suggest that tau burden specifically in EC, IT, IP, and rMFG may be important indicators of disease stage, and indeed longitudinal tau PET changes are higher in these regions among CU A+ and clinically impaired A+ (Harrison et al., 2019; Cho et al., 2019). Thus, we focus on these four a priori regions along with the precentral gyrus, a region that is relatively spared from tau deposition in post-mortem studies (Braak and Braak, 1991; Braak and Del Tredici, 2018; Alafuzoff et al., 2008).

Although amyloid status has been shown to be a predictor of future tau accumulation (Jack et al., 2018), it is unclear whether baseline tau magnitude provides additional information regarding longitudinal change in tau. The few studies that have examined the relation between baseline tau and longitudinal change in tau have yielded inconsistent results. Some studies have shown a positive relation in baseline and future tau accumulation in IT amongst CU (Hanseeuw et al., 2019; Knopman et al., 2020) and in a composite cortical region across the AD continuum (Pontecorvo et al., 2019), whereas others have shown that baseline tau is a non-significant predictor of longitudinal change in a temporal region across the AD continuum (Jack et al., 2020). Thus, baseline predictors of future tau PET accumulation deserve more attention, and it is unclear whether these associations are influenced by reference region and/or disease stage.

Accordingly, this study focuses on how reference region selection specifically influences early tau PET signal during the CU and MCI stages with an emphasis on participants who would be recruited for clinical trials. Our study has four aims: 1) identify group differences (CU A-, CU A+, MCI A+) in cross-sectional and longitudinal change in tau PET in five a priori regions; 2) examine whether current tau burden in these regions predicts annual change in these same regions; 3) conduct exploratory analyses across a large set of regions to determine the regional specificity of our findings; and 4) determine how reference region selection impacts these results.

## 2. Methods

### 2.1. Participants

**Fig. 1.**
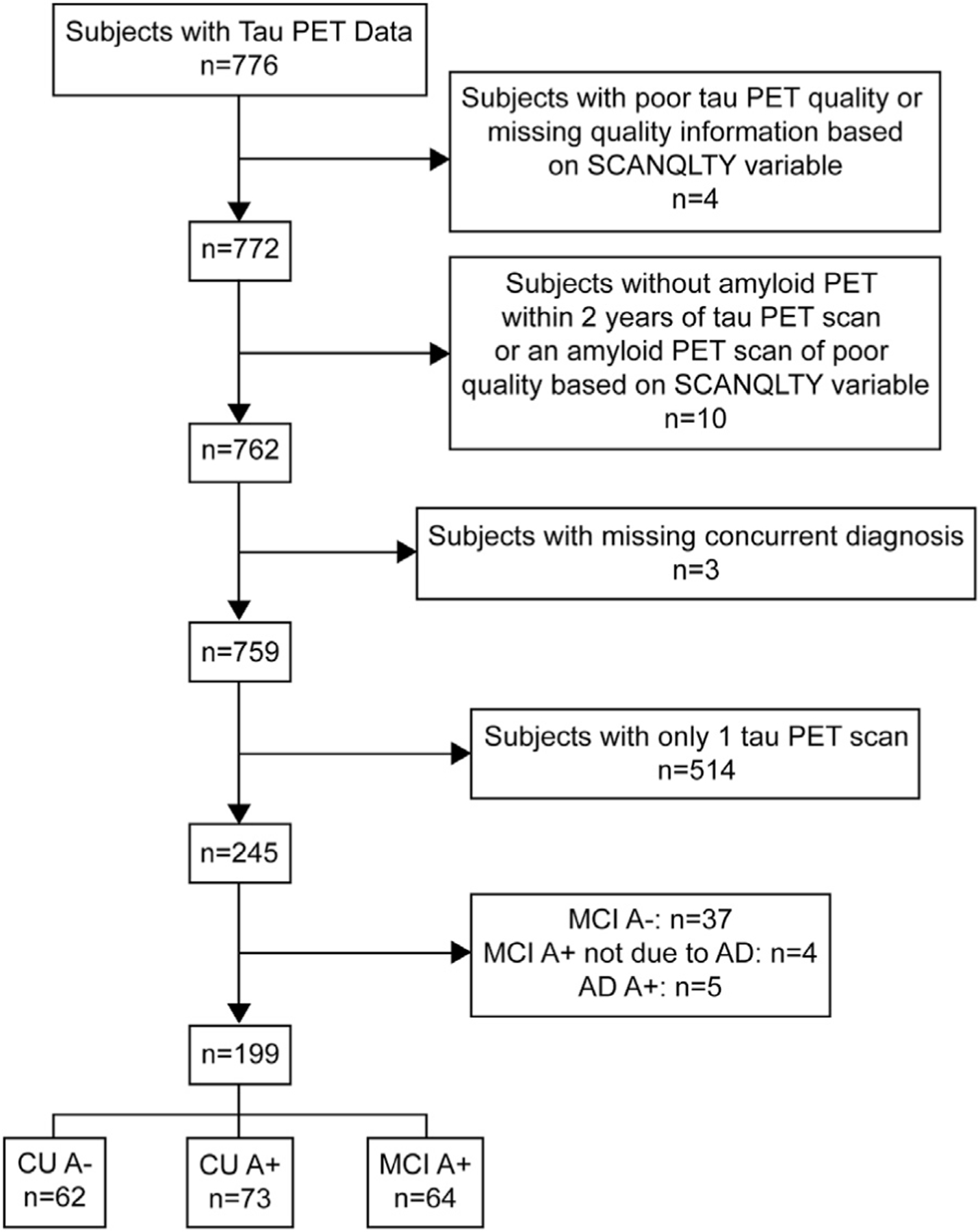
ADNI data selection. CU A- = clinically unimpaired amyloid negative, CU A+ = clinically unimpaired amyloid positive, MCI A+ = mild cognitive impairment amyloid positive.

Data used for this study were obtained from the Alzheimer’s Disease Neuroimaging Initiative (ADNI) database (https://ida.loni.usc.edu). The ADNI was launched in 2003 as a public-private partnership with the primary goal of testing whether serial neuroimaging and biological markers, and clinical and neuropsychological assessment can be combined to measure the progression of MCI and early AD. All ADNI participants provided written informed consent in compliance with local IRBs. For up-to-date information, see www.adni-info.org. For this study, only ADNI participants with ≥ 2 tau PET scans; an amyloid PET within two years of the first tau PET; and a diagnosis of CU or MCI likely due to AD at the time of the first tau PET (mean (SD) = 15 (50) days); and amyloid positivity for those with MCI were included (Fig. 1, Table 1). The time delay between the first and most recent tau PET scans were similar between all groups (Levene’s test: *F*(2,196) = 0.867, *p* = 0.422).

### 2.2. PET imaging

A detailed description of flortaucipir (FTP) standardized uptake value ratio (SUVRs) processing can be found in the ADNI UC Berkeley AV1451 Methods document available on LONI. Regional tau PET measured with FTP for all Freesurfer-defined regions were downloaded from LONI and SUVRs were calculated based on four possible reference regions – (1) eroded subcortical WM, (2) inferior cerebellar GM, (3) a composite consisting of a volume-weighted average of eroded subcortical WM, brainstem, and whole cerebellum, similar to what has been used for examination of amyloid PET over time (Landau et al., 2015; Lowe et al., 2018), and (4) whole cerebellum. All analyses were repeated with partial volume corrected data (Supplementary Figs. 1–5; Supplementary Tables 3–7). Using an amyloid PET closest in time to the first tau PET, individuals with SUVRs (whole cerebellum reference) ≥ 1.08 for [18] F-florbetaben (*n* = 49) or 1.11 for ^[18]^ F-florbetapir (*n* = 150) were considered A+ as suggested by ADNI. Additional details regarding derivation of these cutoffs are available in the Florbetaben and Florbetapir Processing Methods documents available on LONI.

### 2.3. CSF phosphorylated Tau

CSF phosphorylated Tau (pTau-181) processed via the fully automated Elecsys system was examined, converting the 2019 batch to 2016/2017 levels using the regression equation provided by ADNI. pTau-181 levels are expressed in pg/ml. Of the participants included for this study, CSF pTau-181 drawn within one year of the baseline tau PET scan was available for 120/199 participants (32 CU A- (51.6%), 48 CU A+ (65.7%), and 40 MCI A+ (62.5%) participants).

### 2.4. Statistical analyze

Data were analyzed using *R* version 4.0.4. FTP SUVRs were z-score normalized using the mean (SD) from the CU A- group (Supplementary Table 8) to enable comparisons across reference regions. Given the high correlations between FTP SUVRs based on eroded WM and composite, and between inferior cerebellum and whole cerebellum reference regions (see Results), as well as the common use of eroded WM and inferior cerebellum in the literature, subsequent analyses focused on eroded WM and inferior cerebellum reference regions. All models were separately applied for eroded WM and inferior cerebellum reference regions. First, linear regression models examined the effect of group (i.e., CU A-, CU A+, MCI A+) on baseline regional normalized FTP SUVRs (Z(FTP SUVR)), controlling for mean-centered age (74 years). All pairwise contrasts were examined. Second, we used linear regression models using Z(FTP SUVR) across the five a priori regions to predict CSF pTau-181 across the three groups, and to determine if the relation between CSF pTau-181 and regional Z(FTP SUVR) varied by group (SUVR x Group); baseline Z(FTP SUVR) x Group interactions were subsequently removed from the models as these interactions were non-significant across all a priori regions for both reference regions.

Third, to examine change in *Z*(FTP SUVRs) over time, we conducted linear mixed models with fixed effects of Group x Time and Age x Time, and a random intercept; time is the only time-varying term and interactions were not removed if they were non-significant to allow for direct comparisons across all models. Fourth, linear mixed models were applied for each of the five a priori regions (*e.g.*, EC Z(FTP SUVR)_ij_ ∼ *β*_1_ + *β*_2_Baseline Z(FTP SUVR) in EC_i_ + *β*_3_Group_i_ + *β*_4_Age_i_ + *β*_5_Time_ij_ + [*β*_6_Baseline Z(FTP SUVR) in EC_i_ * Time_ij_] + [*β*_7_Baseline Z(FTP SUVR) in EC_i_ * Group_i_] + [*β*_8_Group_i_ * Time_ij_] + [*β*_9_Age_i_ * Time_ij_] + [*β*_10_Group_i_ * Baseline Z(FTP SUVR) in EC_i_ * Time_ij_] + b_1i_ where *i* = participant and *j* = time from first tau scan) to determine whether baseline tau predicted annual change in tau within the same region, and whether the relation between baseline tau and annual change in tau differed by group; non-significant interactions again were not removed to allow for direct comparison across models. For this analysis, the dependent variable was change from baseline *Z*(FTP SUVR) in each region and baseline values (set to zero) were excluded from the outcome but included as an independent predictor. The time term reflects time from first tau PET. Contrasts were examined to determine group differences in longitudinal tau change (*e.g*., does the rate of change *differ across* CU A- and CU A+?), and to determine whether rates of change were significantly different than zero within each group (*e.g*., is the rate of change significantly greater than zero for CU A-?).

Fifth, expanding beyond our a priori regions, FTP SUVRs in 35 Freesurfer regions were z-score normalized using the mean and SD from the CU A- group to enable comparisons of effects sizes across the two different reference regions. Similar to the approach taken for the 5 a priori regions, linear regression models were used to examine the effects of age and group (i.e., CU A-, CU A+, MCI A+) on baseline *Z*(FTP SUVR) for each Freesurfer region. Finally, to understand how reference region influences change in tau, linear mixed models with fixed effect interactions of Group x Time and Age *x* Time, and a random intercept effect were examined; non-significant interactions were not removed from the models to allow for direct comparison across target and reference regions.

## 3. Results

Although we initially examined regional FTP SUVRs based on four possible reference regions (*i.e*., (1) eroded subcortical WM, (2) inferior cerebellar GM, (3) a composite region created using a weighted average of whole cerebellum, brainstem, and eroded subcortical WM, and (4) whole cerebellum), eroded WM and composite reference regions yielded nearly identical FTP SUVRs (range of R^2^ values = 0.874–0.982), as did inferior cerebellum and whole cerebellum reference regions (range of R^2^ values = 0.969–0.990) (Supplementary Fig. 6). Given this redundancy and the fact that eroded WM and inferior cerebellum are more commonly used reference regions in the literature (Hanseeuw et al., 2019; Jack et al., 2018; Knopman et al., 2020; Franzmeier et al., 2020; Vogel et al., 2020; Chiotis et al., 2018; Sintini et al., 2020; Utianski et al., 2020; Sintini et al., 2019; Harrison et al., 2019), subsequent analyses focused on inferior cerebellum and eroded WM, which showed varying coefficients of determination (range of R^2^ values = 0.078–0.800).

**Fig. 2.**
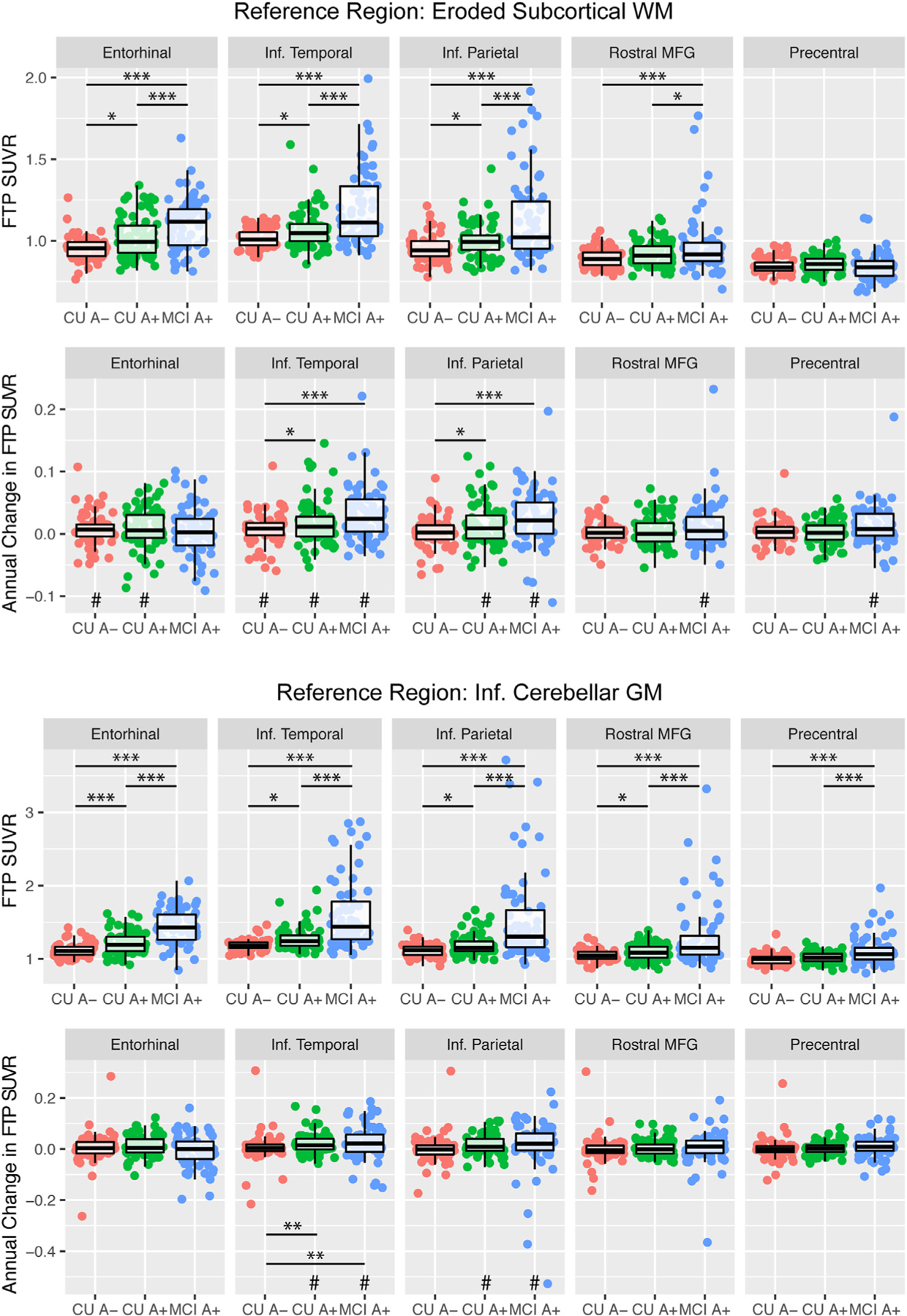
Group differences in baseline flortaucipir (FTP) SUVR and longitudinal change in FTP SUVR based on (A) eroded subcortical white matter (WM) and (B) inferior cerebellar gray matter (GM) reference regions. Age is controlled for in each model. For longitudinal change, annual change in FTP SUVR was manually calculated from a linear mixed model with a random intercept for each Subject and a random slope representing Time for display purposes only; reported statistics are from linear mixed models described in the Methods. * *p* < 0.05, * * *p* < 0.01, * * * *p* < 0.001 for between group contrasts. #*p* < 0.05 for longitudinal change greater than zero within each group.

**Fig. 3.**
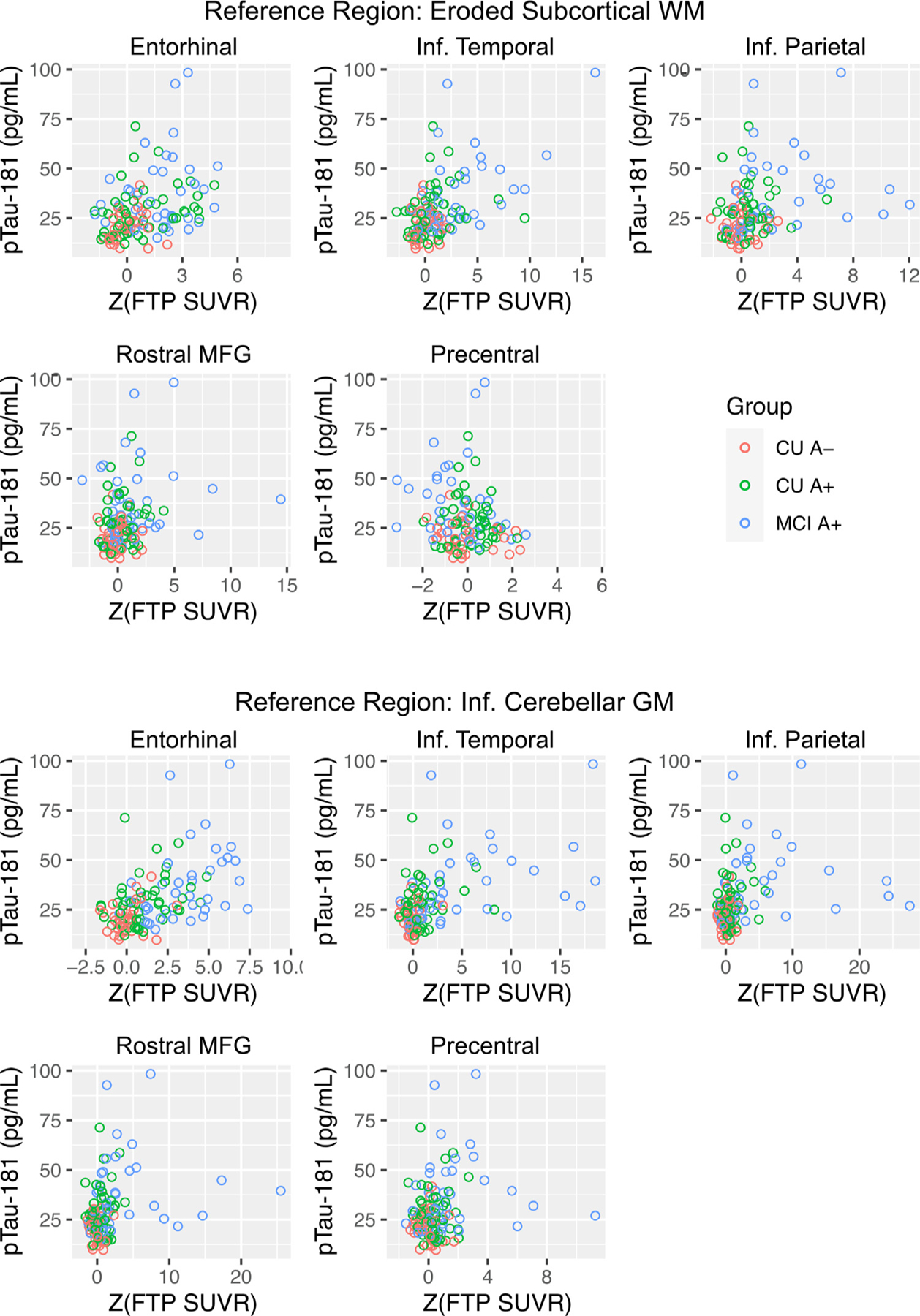
Relation between z-score normalized baseline flortaucipir SUVR (Z(FTP SUVR)) and CSF phosphorylated tau (pTau-181).

Linear regression models controlling for age demonstrated that CU A+ and MCI A+ showed significant baseline elevations in EC, IT, IP, and rMFG regardless of reference region in comparison to CU A- (Fig. 2). With the inferior cerebellum reference region, MCI A+ also showed significant baseline elevations in precentral gyrus. Correlations between regional cross-sectional FTP SUVR and CSF pTau-181 were consistent between reference regions. More specifically, CSF pTau-181 was significantly related to baseline regional FTP SUVR levels in EC, IT, IP, and rMFG with either reference region (Table 2, Fig. 3). CSF pTau-181 was additionally related to precentral FTP SUVR using the inferior cerebellum reference region only. The relation between pTau-181 and regional FTP SUVR did not differ across groups in any region using either reference region (all Baseline FTP SUVR *x* group interactions *p*-values > 0.380) and the Baseline *x* Group interaction terms were thus removed. Overall, a consistent pattern of group differences was present for baseline tau between reference regions with a more extensive pattern of group differences with the inferior cerebellum reference region.

To examine longitudinal tau change, linear mixed models with fixed effects of Group x Time and Age *x* Time, as well as a random intercept were used (Supplementary Table 1). When comparing longitudinal change in *Z*(FTP SUVR) across groups using a Group x Time term in the linear mixed models, there were significant group differences between MCI A+ and CU A+ in comparison to CU A- for IT and IP with the eroded WM reference region (Fig. 2; Supplementary Fig. 7). With the inferior cerebellum reference region, group differences in change in Z(FTP SUVR) were observed in IT only. Significant accumulation greater than zero was present for multiple regions using eroded WM, but only for IT and IP using inferior cerebellum. Overall, this pattern suggests that the eroded WM reference region may be more sensitive to detecting change over time. Further, IT effects were consistently observed across both reference regions for within group changes as well as between group differences indicating that this region may be more robust against methodological decisions such as reference region selection.

We then examined longitudinal change in tau within each group. These results showed that use of the eroded WM reference region revealed significant longitudinal increases in Z(FTP SUVR) for most regions across all three groups. Conversely, change in Z(FTP SUVR) was significantly different than zero only amongst CU A+ and MCI A+ in IT and IP when using the inferior cerebellum reference region (Table 3). More specifically, with the eroded WM reference region, CU groups showed significant increases in EC Z(FTP SUVR) whereas MCI A+ did not; all three groups showed significant increases in Z(FTP SUVR) in IT though the effect was smallest amongst CU A-; both A+ groups showed significant increases in Z(FTP SUVR) in IP whereas CU A- did not; and only MCI A+ showed significant Z(FTP SUVR) increases in rMFG and precentral gyrus. Change in precentral gyrus remained significant even after removal of the one extreme outlier (*B* (SE) = 0.105 (0.041), *p* = 0.011). Overall, this pattern of longitudinal change using the eroded WM reference region follows a hypothesized sequence of changes, with early changes in EC among CU individuals, and cortical changes in CU A+ and MCI A+ (Nelson et al., 2012; Betthauser et al., 2020; Braak and Braak, 1991a).

**Fig. 4.**
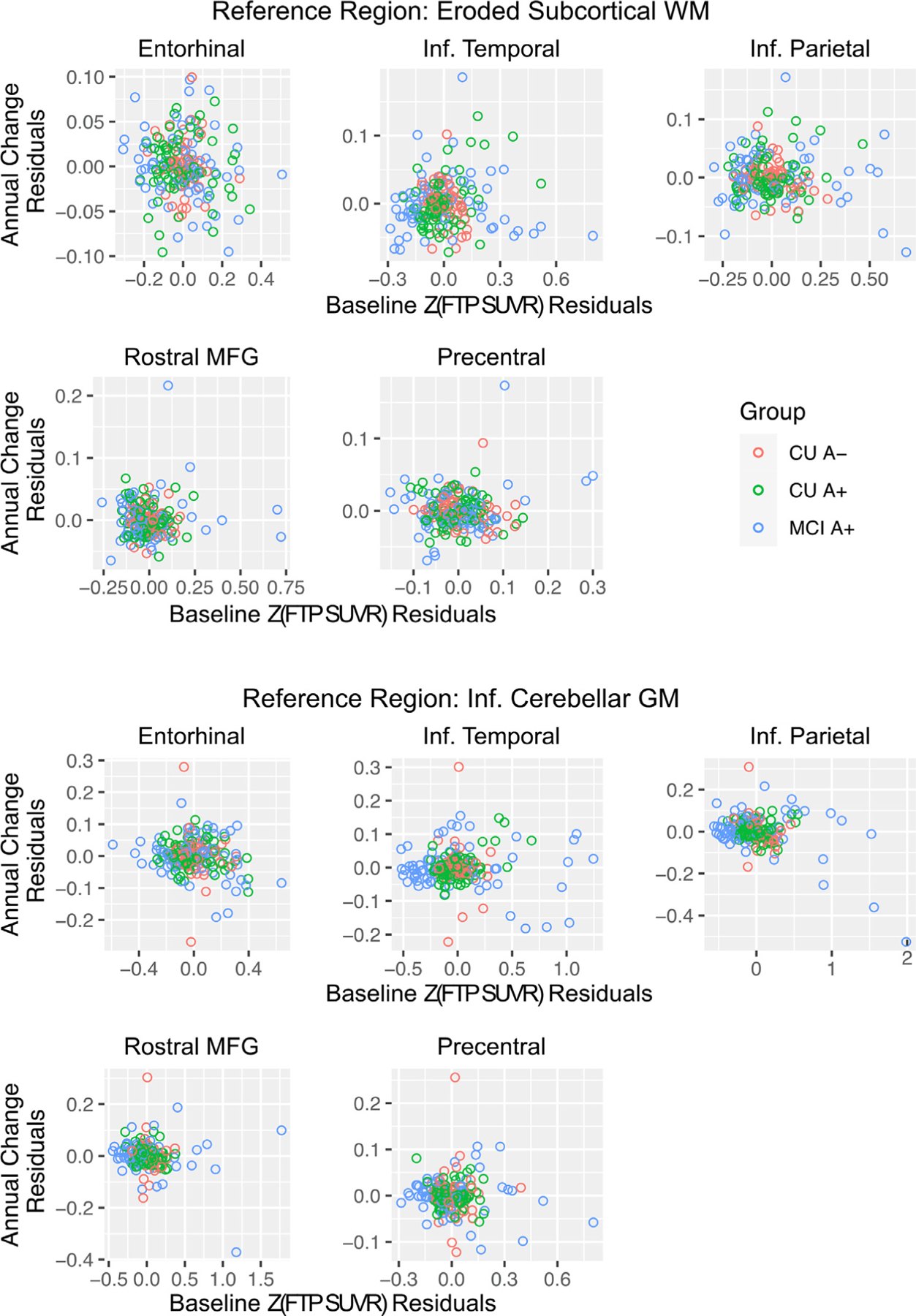
Baseline *z*-score normalized flortaucipir SUVR (Z(FTP SUVR)) versus longitudinal change in Z(FTP SUVR), residualized by age.

Next, we examined whether baseline regional Z(FTP SUVR) levels were associated with longitudinal Z(FTP SUVR) change within the same region with linear mixed models (Supplementary Table 2). In general, baseline Z(FTP SUVR) did not significantly predict change in Z(FTP SUVR) for any group in any region (Table 4, Fig. 4). The two exceptions were for EC for CU A+ with the eroded WM reference region, and baseline predicting change in rMFG for MCI A+ with the inferior cerebellum reference region. When examining whether the relation between baseline and annual change in Z(FTP SUVR) differed by group (*e.g*., Baseline SUVR * CU A+ * Time), CU A+ had a significantly more positive relation when compared to CU A- in EC, IT, and IP; and MCI A+ had a significantly more positive relation when compared to CU A- in IT with the eroded WM reference region; however, these interactions were likely influenced by negative associations within CU A- (higher baseline with lower change) and should be interpreted with caution.

**Fig. 5.**
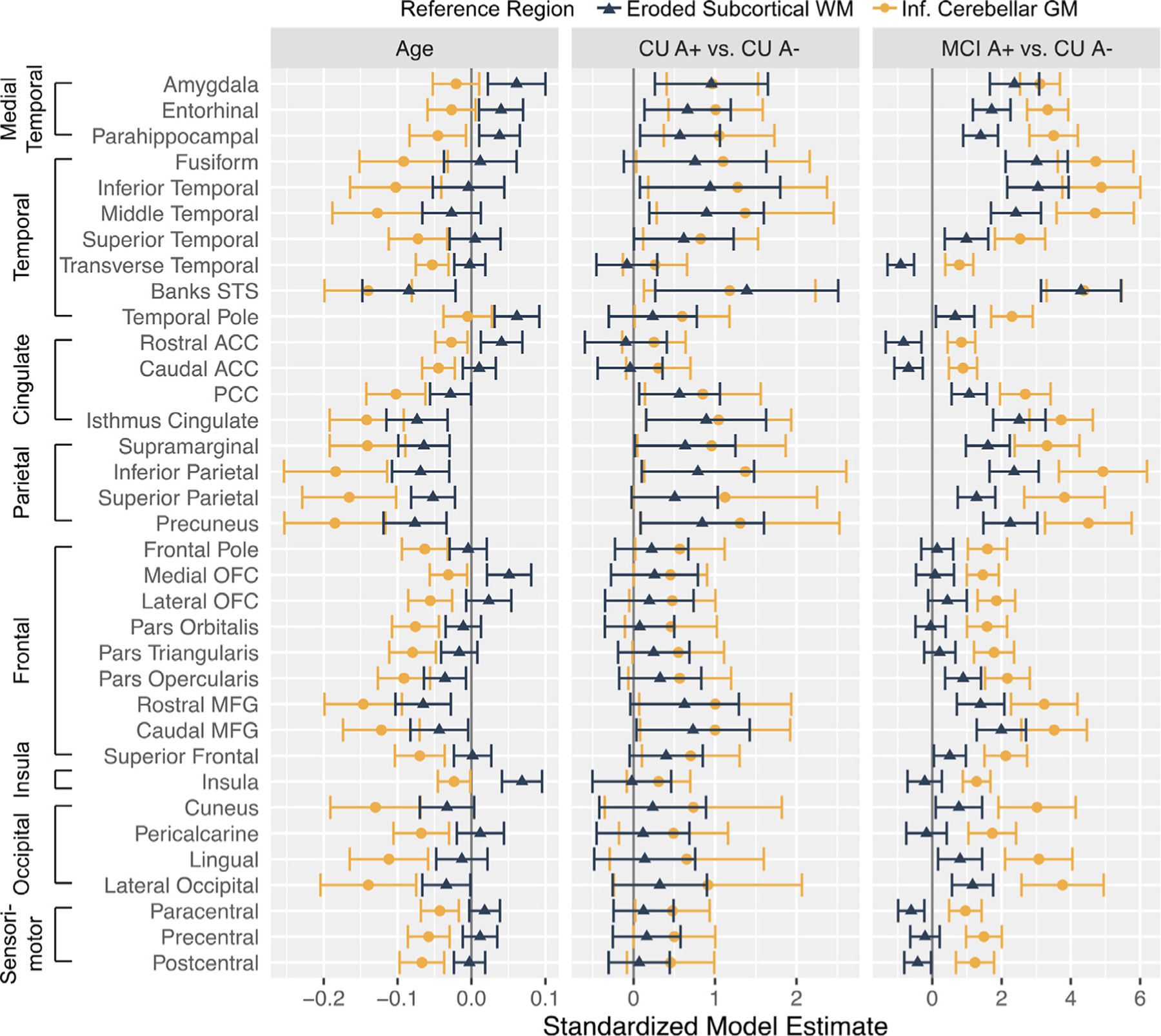
Summary of multiple regression effects of age and group on baseline z-score normalized flortaucipir SUVRs depending on reference region. Error bars depict a 95% confidence interval surrounding the beta estimate.

**Fig. 6.**
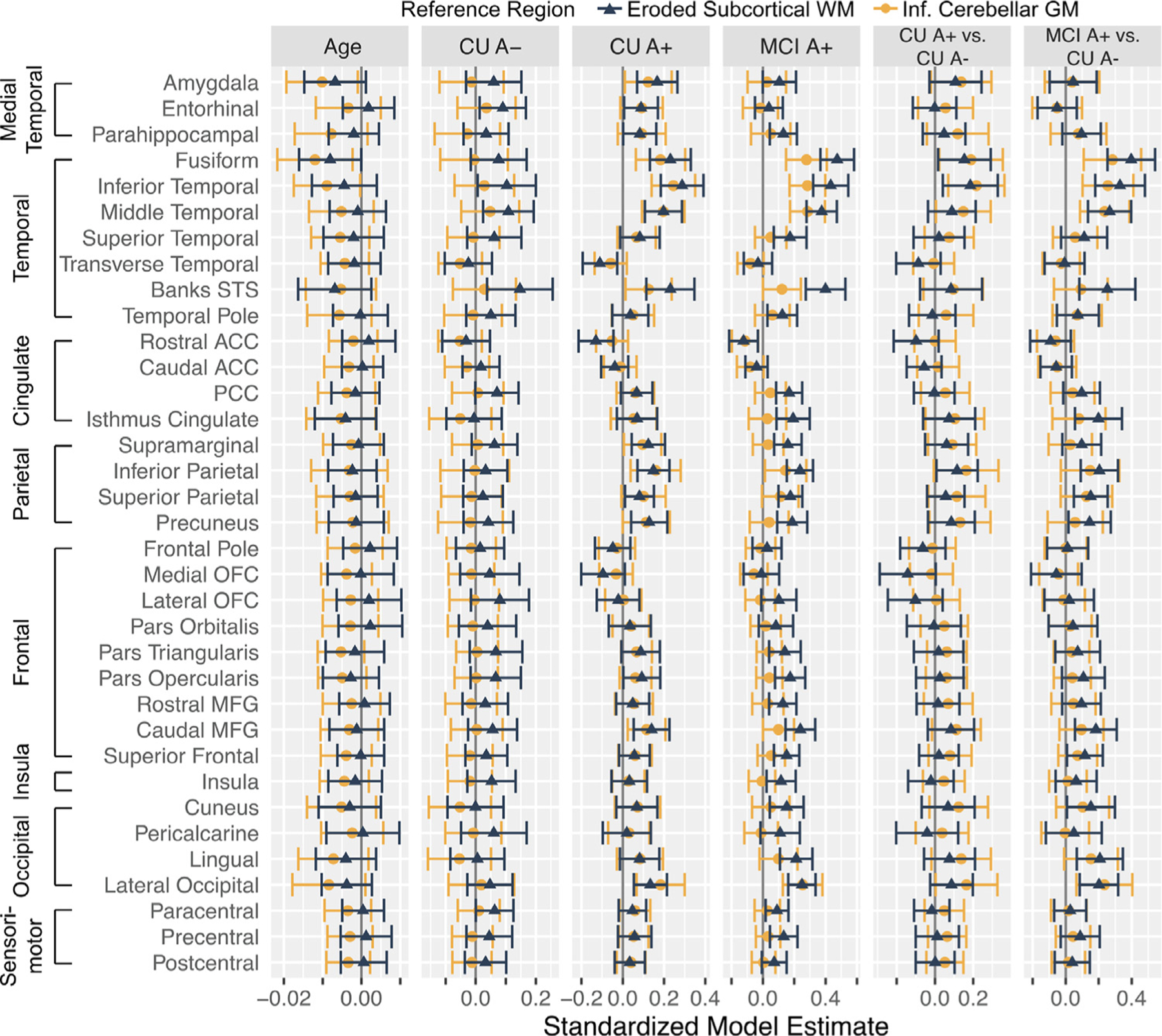
Summary of linear mixed model effects of age and group status on longitudinal z-score normalized flortaucipir SUVRs (Z(FTP SUVR)) depending on reference region. The age column reflects the age x time term (i.e., effect of baseline age on change in Z(FTP SUVR) over time). The CU A-, CU A+, and MCI A+ columns reflect the estimated change in Z(FTP SUVR) for each group (*i.e*., is change in Z(FTP SUVR) different than zero within each group). The CU A+ vs. CU A- and MCI A+ vs. CU A- columns reflect whether change in Z(FTP SUVR) over time differed across groups. Error bars depict a 95% confidence interval.

Given some inconsistencies regarding the spatial pattern of differences between reference regions, we explored the overall pattern of age and group effects using equivalent linear mixed models across 35 Freesurfer regions that are widely used in the literature (Jack et al., 2018) (Fig. 5). This analysis revealed two main baseline differences across reference regions. First, younger age was associated with elevated Z(FTP SUVR) across multiple target regions using the inferior cerebellum reference region. This negative age effect was consistently dampened using the eroded WM reference region. Second, MCI A+ showed significantly elevated Z(FTP SUVR) in all 35 regions relative to CU A- using the inferior cerebellum reference region compared to a more focal pattern of elevations with the eroded WM reference region. When examining longitudinal tau change across the 35 Freesurfer regions (Fig. 6), several regions showed significant change over time within CU A+ and MCI A+, with a tendency for eroded WM to show greater within group accumulation compared to inferior cerebellum.

## 4. Discussion

Our results demonstrated that in regions known to show tau elevations in AD, there are robust baseline differences between CU A-, CU A+, and MCI A+ regardless of reference region. Change over time in IT and IP were also observed regardless of reference region, but changes in EC amongst CU groups and in rMFG amongst MCI A+ were observed only with the eroded WM reference region, suggesting a sequential involvement of these regions. With few exceptions, baseline regional tau was not predictive of longitudinal change within the same region. Exploratory examination across numerous regions revealed a few findings related to reference region choice. For cross-sectional data, use of the inferior cerebellum reference region resulted in stronger negative associations with age and a more distributed pattern of baseline elevations in MCI A+. For longitudinal change, the eroded WM reference region more consistently detected change over time that was significantly different than zero. Overall, these results highlight that baseline and longitudinal accumulation differ across disease stages defined by clinical diagnosis and amyloid status, and that reference region selection influences some of these results. With cross-sectional data, inferior cerebellum may be a more sensitive reference region given that it best distinguishes CU A-, CU A+, and MCI A+ groups. With longitudinal data, eroded WM may be a more sensitive reference region given that this region shows within group change greater than zero in regions consistent with Braak staging.

In accordance with cross-sectional studies that have consistently demonstrated elevated tau among MCI versus CU individuals (Lowe et al., 2018), our results also show greater tau deposition in EC, IT, IP, and rMFG in CU A+ and MCI A+ compared to CU A-. Notably, tau PET elevation in CU A+ and MCI A+, and significant group differences were robust and generally did not depend on reference region. However, analyses using the inferior cerebellum reference region also indicated significantly elevated tau deposition among CU A+ in rMFG as well as among MCI A+ in a distributed set of target regions including precentral gyrus. Validation of cross-sectional tau PET against CSF pTau-181 yielded similar results with associations between pTau-181 and EC, IT, IP, and rMFG SUVRs with either reference region amongst all participants, and an additional relation between pTau-181 and precentral gyrus SUVRs with the inferior cerebellum reference region. Our moderate correlations between pTau-181 and FTP SUVRs are consistent with those reported by Washington University in St. Louis using FTP (Boerwinkle et al., 2021) and BioFinder using [18F]RO-948 (Leuzy et al., 2020). These findings highlight that both reference regions can identify baseline group differences and show expected associations with a different tau biomarker; however, the inferior cerebellum reference region results in additional group distinctions that are less regionally specific.

Our exploratory approach examining regions across the brain also revealed a consistent negative association between age and tau elevations with the inferior cerebellum but not eroded WM reference region. One possibility is that increased tau signal at younger ages reflects more aggressive tau pathology (Schöll et al., 2017; Cho et al., 2017; Pontecorvo et al., 2017). It is also possible that younger *A*+ impaired individuals are more likely to have a “pure” underlying tau pathology (Schöll et al., 2017; Cho et al., 2017), in comparison to older individuals who may have a multifactorial etiology (*e.g*., vascular, alpha synuclein, TDP43) and therefore require less tau accumulation for a given level of impairment. However, dependence on reference region to reveal this negative age association could also imply that an age-related source of off-target binding influences this pattern. For instance, older individuals may be more likely to have age-related mineralization in the cerebellum, which is consistent with known off-target binding of AV1451 in basal ganglia thought to reflect mineralization (Lowe et al., 2016; Harder et al., 2008). Importantly, age-related increases in off-target binding would systematically drive down SUVRs in target regions, giving the appearance of less tau at older ages when in fact reference region signal is elevated. Future studies are needed to understand potential changes related to off-target binding in the cerebellum and WM to guide interpretation of tau PET signal in target regions and negative age associations.

Compared to cross-sectional data, annual tau change was more regionally specific and in some regions, dependent on whether an eroded WM or inferior cerebellum reference region was used. Generally, significant change greater than zero was detected within groups for the eroded WM reference region. More specifically, CU groups, but not MCI, showed similar tau increases greater than zero in EC, and CU *A*+ and MCI *A*+ showed clear increases in IT and IP. This is consistent with patterns of tau uptake in CU *A*+ that transition to MCI (Betthauser et al., 2020). Both IT and IP rates of change showed a stage-dependent pattern such that rates were highest in MCI A+ and CU A+. Finally, significant increases in rMFG tau, a frontal region typically impacted in later disease stages (Braak and Braak, 1991; Braak and Braak, 1995; Braak et al., 2006), were observed only in MCI A+ though rates were comparable between groups. Unexpectedly, MCI A+, as a group, also showed significant tau increases in precentral gyrus, a region that does not show tau deposition until very late in disease (Braak and Braak, 1991; Braak and Del Tredici, 2018; Alafuzoff et al., 2008; Braak and Del Trecidi, 2015), with non-significant differences between groups. However, the vast majority of participants within the MCI A+ group did not show strong increases in precentral gyrus tau (Figs. 2,4, Supplementary Fig. 7) and future studies with post-mortem validation are needed to determine whether this increased signal is reflective of methodologically related confounding factors, particularly given the lower range of values in this region, or regional variability in true tau pathology. In summary, the eroded WM reference region revealed a more focal pattern of longitudinal tau change consistent with our understanding of regional patterns of tau accumulation and spread.

Critically, IT and IP showed significant accumulation over time with both reference regions in CU A+ and MCI A+ groups, suggesting that signal within a subset of target regions may less prone to methodological decisions and more reliable than others. This observation argues against the use of a global composite target region, which is an especially important consideration for clinical trials that are using tau PET as biomarker endpoints. Although tau PET data has been integrated into many clinical trials, to our knowledge, only one study to date has published tau PET data in the context of an anti-amyloid treatment (Mintun et al., 2021). This recent phase 2 trial of donanemab did not show reductions in global tau but did show regional effects in lateral temporal and frontal lobes (Mintun et al., 2021). This further highlights the value of focal target regions for detecting longitudinal change. The donanemab findings in lateral temporal regions converges with our findings in IT tau, and although we did not find consistent accumulation across frontal regions, this discrepancy could be related to differences in cohort characteristics (*e.g*., our study is focused on CU and MCI, whereas the donanemab trial was focused on early symptomatic AD (Mintun et al., 2021)). It is likely that the specific set of regions showing the highest target signal varies according to disease stage, and indeed, our exploratory analyses further revealed frontal and occipital changes in MCI A+ only with the eroded WM reference region. In contrast, the inferior cerebellum reference region yielded change estimates around zero in 28/35 brain regions in MCI A+, a stage in which tau change is expected to play a significant role (Jack et al., 2010). Taken together, consideration of target regions as well as reference region selection may be especially important to consider for detecting change amongst those with early cognitive impairment.

Few studies with conflicting results have examined whether baseline tau load is an important predictor of subsequent accumulation (Hanseeuw et al., 2019; Knopman et al., 2020; Pontecorvo et al., 2019; Jack et al., 2020). Our findings here indicate that baseline tau generally does not significantly predict change in future tau within the same region after accounting for disease stage. It is noteworthy however that CU A+ showed consistent positive associations between baseline and change in tau in all target regions with either reference region, whereas associations within the higher range of tau values among the MCI A+ group were largely uncoupled. It is possible that with a larger sample size and longer follow-up time, a clearer association between baseline and change in tau amongst CU A+ will be identified. Furthermore, qualitative examination of these relations revealed that individuals with the highest baseline values (MCI A+) do not show the greatest accumulation within that region. This could reflect an uncoupling between magnitude and rate indicating saturation of PET signal and/or lack of further tangle deposition in regions with significant tangle pathology. Overall, our results suggest that longitudinal change in regional tau is related to disease stage and amyloid status (Hanseeuw et al., 2019; Knopman et al., 2020; Vogel et al., 2020; Pontecorvo et al., 2019; Guo et al., 2020; Chen et al., 2020) and that higher baseline levels of tau do not directly translate to greater change slopes in those same regions.

There are several limitations that are important to consider. The majority of participants only had two timepoints and follow-up was limited to 1.6 years (mean). Longer follow-up and additional timepoints may reveal important nonlinear associations with tau change, especially among CU. Second, given our focused a priori hypotheses, our statistical models did not correction for multiple comparisons. Third, factors such as age or disease associated perfusion can differentially affect SUVR quantification between reference regions, which could account for the differences observed here. Fourth, the SUVR data that is provided by ADNI did not use longitudinal processing or within-person templates for segmentation. These processing decisions may influence interpretations of regional SUVRs, particularly for subjects with significant atrophy or structural abnormalities. We also cannot compare our results to gold-standard dynamic data with arterial sampling, which have not explored white matter as a potential reference region and have rather used cerebellum given the low bias and high consistency of binding in this region (Barret et al., 2017; Wooten et al., 2017; Hahn et al., 2017). However, the data presented here are similar to what is feasible in a clinical trial setting.

## 5. Conclusions

Our study showed an uncoupling between regional baseline tau and changes in tau, and indicated that reference region selection can influence the effect of age on baseline tau, regional specificity of baseline group differences, and the ability to detect significant change over time. Inferior cerebellar gray matter may be a preferable reference region for cross-sectional flortaucipir focused on group differences, whereas eroded subcortical white matter may be a preferable reference region for longitudinal flortaucipir analyze examining regional patterns of change.

## Supplementary Material

SupplementaryMaterials

## Figures and Tables

**Table 1:** Participant demographics at the first tau PET scan. Statistics refer to differences between CU A−, CU A+, and MCI A+ groups.

Characteristic	CU A− (*n* = 62)	CU A+ (*n* = 73)	MCI A+ (*n* = 64)	All (*n* = 199)	Statistics
Age, years					*F*(2,196) = 4.28, *p* = 0.015 *
Mean (Range)	72 (58–90)	75 (62–90)	75 (56–92)	74 (56–92)	
Sex, n (%)					*χ* ^*2*^ (2) = 2.13, *p* = 0.345
Female	34 (55)	40 (55)	28 (44)	102 (51)	
Male	28 (45)	33 (45)	36 (56)	97 (49)	
Education, years					*F*(2,196) = 2.00, *p* = 0.138
Mean (Range)	17 (12–20)	17 (12–20)	16 (12–20)	16 (12–20)	
Ethnicity, n (%)					*χ* ^*2*^ (4) = 6.04, *p* = 0.196
Hispanic/Latino	5 (8)	2 (3)	2 (3)	9 (5)	
Not Hispanic/Latino	57 (92)	69 (95)	62 (97)	188 (94)	
Unknown	0 (0)	2 (3)	0 (0)	2 (1)	
Race, n (%)					*χ* ^*2*^ (6) = 9.86, *p* = 0.131
American Indian/Alaskan Native	1 (2)	0 (0)	0 (0)	1 (1)	
Asian	0 (0)	0 (0)	0 (0)	0 (0)	
Native Hawaiian/Other Pacific Islander	0 (0)	0 (0)	0 (0)	0 (0)	
Black/African American	4 (6)	3 (4)	0 (0)	7 (4)	
White	54 (87)	70 (96)	61 (95)	185 (93)	
More than 1 Race	3 (5)	0 (0)	3 (5)	6 (3)	
Amyloid PET, SUVR					*F*(2,196) = 124.60, *p* < *0*.001 * * *
AV45 Mean (95% CI)	1.02 (1.01–1.03)	1.33 (1.28–1.38)	1.38 (1.13–1.43)	1.24 (1.21–1.27)	
FBB Mean (95% CI)	0.98 (0.97–0.99)	1.37 (1.29–1.45)	1.55 (1.45–1.65)	1.35 (1.27–1.43)	
Tau PET Scans, n					*F*(2,196) = 0.36, *p* =0.698
Mean (Range)	2 (2–5)	2 (2–4)	2 (2–4)	2 (2–5)	
Total Tau Scan Interval, years					*F*(2,196) = 2.67, *p* =0.072
Mean (95% CI)	1.8 (1.57–1.95)	1.6 (1.41–1.71)	1.5 (1.28–1.65)	1.6 (1.49–1.69)	

**Table 2:** Relation between z-score normalized baseline flortaucipir SUVR (Z(FTP SUVR)) and CSF phosphorylated tau (pTau-181) amongst CU A−, CU A+, and MCI A+ groups.

	Ref. Region: Eroded Subcortical WM		Ref. Region: Inf. Cerebellar GM	
	*B* (SE)	*p*	*B* (SE)	*p*
Baseline EC	**3.588 (0.799)**	***<* 0.001** ***	**3.722 (0.561)**	***<* 0.001** ***
Baseline IT	**2.846 (0.417)**	***<* 0.001** ***	**1.808 (0.301)**	***<* 0.001** ***
Baseline IP	**1.902 (0.558)**	***<* 0.001** ***	**0.908 (0.280)**	**0.002** **
Baseline rMFG	**1.645 (0.655)**	**0.013** *	**1.139 (0.380)**	**0.003** **
Baseline Precentral	−2.108 (1.279)	0.102	**1.802 (0.827)**	**0.031** *

EC = entorhinal cortex, IT = inferior temporal, IP = inferior parietal, rMFG = rostral middle frontal gyrus

*** *p* < 0.001

** *p* < 0.01

* *p* < 0.05

† *p* < 0.10.

**Table 3:** Longitudinal change in *z*-score normalized flortaucipir SUVR (Z(FTP SUVR)) within each group. Age and Age x Time are included in each model.

	Ref. Region: Eroded Subcortical WM		Ref. Region: Inf. Cerebellar GM	
	*B* (SE)	*p*	*B* (SE)	*p*
Longitudinal Change in Z(FTP SUVR) vs. 0				
Entorhinal				
CU A−	**0.090 (0.039)**	**0.022** *	0.036 (0.049)	0.470
CU A+	**0.088 (0.042)**	**0.035** *	0.091 (0.053)	0.085 †
MCI A+	0.038 (0.045)	0.396	−0.016 (0.057)	0.780
Inferior Temporal				
CU A−	**0.103 (0.049)**	**0.037** *	0.028 (0.051)	0.573
CU A+	**0.288 (0.053)**	***<* 0.001** ***	**0.245 (0.054)**	***<* 0.001** ***
MCI A+	**0.433 (0.057)**	***<* 0.001** ***	**0.284 (0.058)**	***<* 0.001** ***
Inferior Parietal				
CU A−	0.033 (0.037)	0.377	−0.003 (0.058)	0.954
CU A+	**0.149 (0.040)**	***<* 0.001** ***	**0.159 (0.062)**	**0.010** *
MCI A+	**0.237 (0.043)**	***<* 0.001** ***	**0.144 (0.067)**	**0.031** *
Rostral Middle Frontal Gyrus				
CU A−	0.032 (0.039)	0.412	−0.015 (0.044)	0.727
CU A+	0.048 (0.041)	0.246	0.054 (0.047)	0.251
MCI A+	**0.127 (0.045)**	**0.004** **	0.032 (0.051)	0.531
Precentral Gyrus				
CU A−	0.045 (0.039)	0.246	−0.012 (0.034)	0.738
CU A+	0.057 (0.041)	0.169	0.052 (0.037)	0.156
MCI A+	**0.133 (0.045)**	**0.003** **	0.033 (0.040)	0.403

**Table 4:** Effects of z-score normalized baseline flortaucipir SUVR (Z(FTP SUVR)) predicting longitudinal change in Z(FTP SUVR). Age is controlled for in each model. Contrasts are shown to summarize the association between baseline Z(FTP SUVR) and change in Z(FTP SUVR) over time within each group (Baseline Z(FTP SUVR) *x* Time), and to determine whether the association between baseline and change in Z(FTP SUVR) varied across groups (Baseline Z(FTP SUVR) *x* Time *x* Group).

	Ref. Region: Eroded Subcortical WM		Ref. Region: Inf.Cerebellar GM	
	*B* (SE)	*p*	*B* (SE)	*p*
Baseline EC Predicting Annual Change in EC				
Baseline Z(FTP SUVR) x Time Within Groups				
CU A−	−0.121 (0.068)	0.075 †	0.075 (0.125)	0.551
CU A+	**0.147 (0.060)**	**0.015** *	0.131 (0.096)	0.173
MCI A+	−0.061 (0.046)	0.182	0.023 (0.053)	0.660
Baseline Z(FTP SUVR) x Time x Group Interactions				
CU A+ vs. CU A−	**0.268 (0.090)**	**0.003** **	0.056 (0.158)	0.723
MCI A+ vs. CU A−	0.060 (0.085)	0.480	−0.051 (0.138)	0.711
Baseline IT Predicting Annual Change in IT				
Baseline Z(FTP SUVR) x Time Within Groups				
CU A−	−0.150 (0.085)	0.077 †	0.237 (0.132)	0.072 †
CU A+	0.054 (0.048)	0.263	0.045 (0.062)	0.469
MCI A+	0.084 (0.060)	0.161	0.020 (0.042)	0.635
Baseline Z(FTP SUVR) x Time x Group Interactions				
CU A+ vs. CU A−	**0.204 (0.097)**	**0.038** *	−0.192 (0.146)	0.190
MCI A+ vs. CU A−	**0.234 (0.107)**	**0.030** *	−0.217 (0.139)	0.120
Baseline IP Predicting Annual Change in IP				
Baseline Z(FTP SUVR) *x* Time Within Groups				
CU A−	−0.097 (0.064)	0.128	0.114 (0.106)	0.285
CU A+	0.074 (0.058)	0.206	0.090 (0.065)	0.164
MCI A+	−0.026 (0.067)	0.699	0.000 (0.042)	0.996
Baseline Z(FTP SUVR) x Time x Group Interactions				
CU A+ vs. CU A−	**0.171 (0.086)**	**0.049** *	−0.024 (0.124)	0.848
MCI A+ vs. CU A−	0.072 (0.095)	0.451	−0.114 (0.114)	0.321
Baseline rMFG Predicting Annual Change in rMFG				
Baseline Z(FTP SUVR) x Time Within Groups				
CU A−	−0.112 (0.075)	0.137	0.080 (0.081)	0.325
CU A+	0.007 (0.062)	0.909	0.068 (0.057)	0.233
MCI A+	0.006 (0.035)	0.865	**0.071 (0.023)**	**0.003** **
Baseline Z(FTP SUVR) x Time x Group Interactions				
CU A+ vs. CU A−	0.119 (0.097)	0.223	−0.012 (0.099)	0.907
MCI A+ vs. CU A−	0.118 (0.083)	0.161	−0.009 (0.084)	0.913
Baseline Precentral Predicting Annual Change in Precentral				
Baseline Z(FTP SUVR) x Time Within Groups				
CU A−	−0.096 (0.066)	0.142	0.046 (0.074)	0.537
CU A+	−0.010 (0.088)	0.911	0.039 (0.069)	0.576
MCI A+	−0.059 (0.096)	0.536	0.016 (0.042)	0.707
Baseline Z(FTP SUVR) x Time x Group Interactions				
CU A+ vs. CU A−	0.086 (0.109)	0.430	−0.007 (0.101)	0.943
MCI A+ vs. CU A−	0.037 (0.118)	0.755	−0.030 (0.085)	0.722

## Data Availability

Data used for this study were obtained from the Alzheimer’s Disease Neuroimaging Initiative (ADNI) database (https://ida.loni.usc.edu). The ADNI was launched in 2003 as a public-private partnership with the primary goal of testing whether serial neuroimaging and biological markers, and clinical and neuropsychological assessment can be combined to measure the progression of MCI and early AD. All ADNI participants provided written informed consent in compliance with local IRBs. For up-to-date information, see www.adni-info.org. Data were analyzed using *R* version 4.0.4.
