# Supplementary material for: Influence of common reference regions on regional tau patterns in cross-sectional and longitudinal [18F]-AV-1451 PET data
Source: Neuroimage. Author manuscript; Available in PMC 2022 Jan 24. (PMC8785682; doi:10.1016/j.neuroimage.2021.118553)
Supplement: SupplementaryMaterials [file NIHMS1771427-supplement-SupplementaryMaterials.docx]

**Supplementary Materials**

**Methods**

All analyses were repeated with partial volume correction (PVC) data using hemispheric white matter and inferior cerebellar gray matter as reference regions. Hemispheric white matter includes all cerebral white matter and was not eroded, whereas the eroded subcortical white matter reference region that was used for the primary non-PVC analyses in the main text included eroded white matter throughout the brain. All data were downloaded from LONI.

**
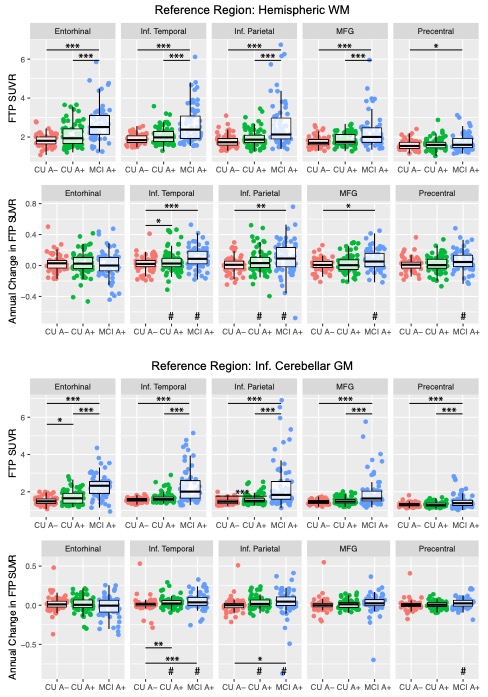
**

**Supplementary Figure 1.** **Group differences in baseline flortaucipir SUVR (FTP SUVR) and longitudinal change in FTP SUVR based on (A) hemispheric white matter (WM) and (B) inferior cerebellar gray matter (GM) reference regions with partial volume correction.** Age is controlled for in each model. For longitudinal analyses, annual change in FTP SUVR was manually calculated from a linear mixed model with a random intercept for each Subject and a random slope representing Time for display purposes only; reported statistics are from linear mixed models described in the Methods. **p*<.05, ***p*<.01, ****p*<.001 for between group contrasts. #*p*<.05 for longitudinal change greater than zero within each group.

**Supplementary Figure 2. Relation between CSF phosphorylated tau (pTau-181) and z-score normalized baseline flortaucipir SUVR (Z(FTP SUVR)) with partial volume correction.**

**Supplementary Figure 3. Baseline z-score normalized flortaucipir SUVR (Z(FTP SUVR)) versus longitudinal change in Z(FTP SUVR) with partial volume correction, residualized by age.**

 **Supplementary Figure 4. Summary of multiple regression effects of age and group on baseline z-score normalized flortaucipir SUVRs with partial volume correction depending on reference regions.** Error bars depict a 95% confidence interval surrounding the beta estimate for each effect.

 **Supplementary Figure 5. Summary of linear mixed model effects of age and group status on longitudinal z-score normalized flortaucipir SUVRs (Z(FTP SUVR)) with partial volume correction depending on reference region.** The age column reflects the age x time term (i.e., the effect of baseline age on change in Z(FTP SUVR) over time). The CU A-, CU A+, and MCI A+ columns reflect the estimated change in Z(FTP SUVR) for each group (i.e., is change in Z(FTP SUVR) different than zero within each group). The CU A+ vs. CU A- and MCI A+ vs. CU A- columns reflect whether change in Z(FTP SUVR) over time differed across groups. Note: Error bars depict a 95% confidence interval.

**Supplementary Figure 6. Flortaucipir SUVRs using whole cerebellum, inferior cerebellar gray matter, composite, and eroded subcortical white matter reference region.**

**Supplementary Figure 7. Spaghetti plot showing change in flortaucipir (FTP) SUVR over time for each participant.**

**
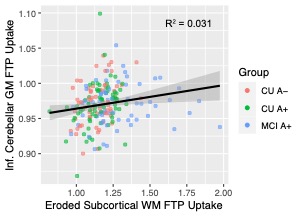
**

**Supplementary Figure 8. Relation between flortaucipir (FTP) uptake in inferior cerebellar gray matter and eroded subcortical white matter/hemispheric white matter regions, after the iterative global normalization procedure implemented by the ADNI PET Core. These values were used to normalize target regions values to determine SUVR.**

**Supplementary Table 1. Longitudinal change in z-score normalized flortaucipir SUVR (Z(FTP SUVR)) with fixed effects of Age, Time, Group, Age x Time, and Group x Time.** Note: *** *p*<.001, ** *p*<.01, * *p*<.05, ^†^ *p*<.10.

|  | | Ref. Region: Eroded Subcortical WM | | Ref. Region: Inf. Cerebellum | |
| --- | --- | --- | --- | --- | --- |
|  | *B* (SE) | | *p* | *B* (SE) | *p* |
| Entorhinal | | | | | |
| Age | 0.039 (0.015) | | **0.011 *** | -0.024 (0.017) | 0.157 |
| Time | 0.090 (0.039) | | **0.022 *** | 0.036 (0.049) | 0.471 |
| CU A+ vs. CU A- | 0.666 (0.274) | | **0.016 *** | 0.986 (0.299) | **0.001 **** |
| MCI A+ vs. CU A- | 1.722 (0.281) | | **<0.001 ***** | 3.324 (0.306) | **<0.001 ***** |
| Age * Time | 0.002 (0.003) | | 0.588 | -0.003 (0.004) | 0.426 |
| CU A+ * Time | -0.002 (0.058) | | 0.976 | 0.055 (0.073) | 0.452 |
| MCI A+ * Time | -0.052 (0.061) | | 0.395 | -0.052 (0.077) | 0.501 |
| Inferior Temporal | | | | | |
| Age | -0.005 (0.026) | | 0.846 | **-0.099 (0.032)** | **0.002 **** |
| Time | 0.103 (0.049) | | **0.038 *** | 0.028 (0.051) | 0.574 |
| CU A+ vs. CU A- | 0.962 (0.460) | | **0.038 *** | **1.263 (0.572)** | **0.028 *** |
| MCI A+ vs. CU A- | 3.074 (0.472) | | **<0.001 ***** | **4.877 (0.587)** | **<0.001 ***** |
| Age * Time | -0.004 (0.004) | | 0.300 | **-0.009 (0.004)** | **0.042 *** |
| CU A+ * Time | 0.185 (0.073) | | **0.012 *** | **0.217 (0.075)** | **0.004 **** |
| MCI A+ * Time | 0.329 (0.077) | | **<0.001***** | **0.256 (0.078)** | **0.001 **** |
| Interior Parietal | | | | | |
| Age | -0.067 (0.020) | | **0.001 **** | **-0.176 (0.034)** | **<0.001 ***** |
| Time | 0.033 (0.037) | | 0.378 | -0.003 (0.058) | 0.954 |
| CU A+ vs. CU A- | 0.828 (0.358) | | **0.022 *** | **1.379 (0.614)** | **0.026 *** |
| MCI A+ vs. CU A- | 2.385 (0.367) | | **<0.001 ***** | **4.908 (0.630)** | **<0.001 ***** |
| Age * Time | -0.002 (0.003) | | 0.464 | -0.003 (0.005) | 0.537 |
| CU A+ * Time | 0.116 (0.055) | | **0.036 *** | 0.163 (0.086) | 0.061 ^†^ |
| MCI A+ * Time | 0.204 (0.057) | | **<0.001 ***** | 0.148 (0.090) | 0.102 |
| Rostral Middle Frontal Gyrus | | | | | |
| Age | -0.068 (0.020) | | **<0.001 ***** | -0.145 (0.027) | **<0.001 ***** |
| Time | 0.032 (0.039) | | 0.413 | -0.015 (0.044) | 0.728 |
| CU A+ vs. CU A- | 0.651 (0.350) | | 0.064 ^†^ | 0.994 (0.474) | **0.037 *** |
| MCI A+ vs. CU A- | 1.440 (0.359) | | **<0.001 ***** | 3.239 (0.486) | **<0.001 ***** |
| Age * Time | 0.001 (0.003) | | 0.810 | -0.003 (0.004) | 0.505 |
| CU A+ * Time | 0.016 (0.057) | | 0.778 | 0.069 (0.065) | 0.290 |
| MCI A+ * Time | 0.095 (0.060) | | 0.112 | 0.047 (0.068) | 0.490 |
| Precentral Gyrus | | | | | |
| Age | 0.009 (0.013) | | 0.464 | -0.056 (0.015) | **<0.001 ***** |
| Time | 0.045 (0.039) | | 0.247 | -0.011 (0.034) | 0.739 |
| CU A+ vs. CU A- | 0.170 (0.227) | | 0.454 | 0.480 (0.261) | 0.070 ^†^ |
| MCI A+ vs. CU A- | -0.164 (0.232) | | 0.481 | 1.510 (0.268) | **<0.001 ***** |
| Age * Time | 0.001 (0.003) | | 0.719 | -0.003 (0.003) | 0.324 |
| CU A+ * Time | 0.012 (0.058) | | 0.835 | 0.064 (0.051) | 0.214 |
| MCI A+ * Time | 0.088 (0.060) | | 0.144 | 0.045 (0.053) | 0.403 |

**Supplementary Table 2. Baseline z-score normalized flortaucipir SUVR (Z(FTP SUVR)) predicting longitudinal change in Z(FTP SUVR).**

|  | | Ref. Region: Eroded Subcortical WM | | Ref. Region: Inf.  Cerebellar GM | |
| --- | --- | --- | --- | --- | --- |
|  | *B* (SE) | | *p* | *B* (SE) | *p* |
| Baseline EC Predicting Annual Change in EC | | | | | |
| Baseline Z(FTP SUVR) | 0.154 (0.129) | | 0.234 | -0.214 (0.218) | 0.327 |
| Age | -0.003 (0.011) | | 0.796 | 0.015 (0.014) | 0.280 |
| Time | 0.044 (0.078) | | 0.574 | -0.001 (0.102) | 0.992 |
| CU A+ vs. CU A- | -0.037 (0.200) | | 0.855 | -0.023 (0.266) | 0.932 |
| MCI A+ vs. CU A- | 0.004 (0.222) | | 0.985 | 0.251 (0.332) | 0.450 |
| Baseline Z(FTP SUVR) * Time | -0.121 (0.068) | | 0.077 ^†^ | 0.075 (0.125) | 0.552 |
| Baseline Z(FTP SUVR) * CU A+ | **-0.344 (0.159)** | | **0.032 *** | 0.027 (0.259) | 0.918 |
| Baseline Z(FTP SUVR) * MCI A+ | -0.148 (0.149) | | 0.322 | 0.098 (0.236) | 0.679 |
| Time * Age | 0.004 (0.007) | | 0.557 | -0.012 (0.009) | 0.195 |
| Time * CU A+ | 0.020 (0.118) | | 0.863 | 0.054 (0.159) | 0.733 |
| Time * MCI A+ | 0.039 (0.132) | | 0.766 | -0.032 (0.185) | 0.863 |
| Baseline Z(FTP SUVR) * CU A+ * Time | **0.268 (0.090)** | | **0.003 **** | 0.056 (0.158) | 0.723 |
| Baseline Z(FTP SUVR) * MCI A+ * Time | 0.060 (0.085) | | 0.480 | -0.051 (0.138) | 0.711 |
| Baseline IT Predicting Annual Change in IT | | | | | |
| Baseline Z(FTP SUVR) | 0.054 (0.162) | | 0.738 | -0.348 (0.219) | 0.114 |
| Age | -0.018 (0.013) | | 0.189 | 0.021 (0.013) | 0.095 ^†^ |
| Time | 0.107 (0.091) | | 0.246 | -0.011 (0.087) | 0.901 |
| CU A+ vs. CU A- | 0.048 (0.241) | | 0.842 | -0.234 (0.245) | 0.341 |
| MCI A+ vs. CU A- | 0.256 (0.265) | | 0.337 | 0.121 (0.266) | 0.650 |
| Baseline Z(FTP SUVR) * Time | -0.150 (0.085) | | 0.080 ^†^ | 0.237 (0.132) | 0.074 ^†^ |
| Baseline Z(FTP SUVR) * CU A+ | 0.052 (0.180) | | 0.774 | 0.510 (0.240) | **0.035 *** |
| Baseline Z(FTP SUVR) * MCI A+ | -0.142 (0.182) | | 0.435 | 0.307 (0.225) | 0.175 |
| Time * Age | 0.006 (0.008) | | 0.452 | -0.020 (0.008) | **0.008 **** |
| Time * CU A+ | 0.072 (0.141) | | 0.613 | 0.218 (0.142) | 0.129 |
| Time * MCI A+ | 0.153 (0.158) | | 0.337 | 0.225 (0.153) | 0.144 |
| Baseline Z(FTP SUVR) * CU A+ * Time | 0.204 (0.097) | | **0.038 *** | -0.192 (0.146) | 0.190 |
| Baseline Z(FTP SUVR) * MCI A+ * Time | 0.234 (0.107) | | **0.030 *** | -0.217 (0.139) | 0.120 |
| Baseline IP Predicting Annual Change in IP | | | | | |
| Baseline Z(FTP SUVR) | 0.002 (0.133) | | 0.988 | -0.281 (0.207) | 0.176 |
| Age | 0.010 (0.010) | | 0.320 | 0.025 (0.013) | 0.054 ^†^ |
| Time | 0.100 (0.067) | | 0.138 | 0.017 (0.078) | 0.828 |
| CU A+ vs. CU A- | 0.254 (0.181) | | 0.162 | 0.078 (0.244) | 0.747 |
| MCI A+ vs. CU A- | 0.113 (0.196) | | 0.564 | 0.402 (0.256) | 0.117 |
| Baseline Z(FTP SUVR) * Time | -0.097 (0.064) | | 0.131 | 0.114 (0.106) | 0.287 |
| Baseline Z(FTP SUVR) * CU A+ | -0.002 (0.160) | | 0.991 | 0.321 (0.233) | 0.170 |
| Baseline Z(FTP SUVR) * MCI A+ | 0.019 (0.157) | | 0.904 | 0.179 (0.212) | 0.400 |
| Time * Age | -0.009 (0.007) | | 0.181 | -0.024 (0.007) | **<0.001 ***** |
| Time * CU A+ | -0.052 (0.106) | | 0.624 | 0.057 (0.132) | 0.666 |
| Time * MCI A+ | 0.186 (0.116) | | 0.111 | 0.205 (0.134) | 0.128 |
| Baseline Z(FTP SUVR) * CU A+ * Time | 0.171 (0.086) | | **0.049 *** | -0.024 (0.124) | 0.848 |
| Baseline Z(FTP SUVR) * MCI A+ * Time | 0.072 (0.095) | | 0.451 | -0.114 (0.114) | 0.321 |
| Baseline rMFG Predicting Annual Change in rMFG | | | | | |
| Baseline Z(FTP SUVR) | 0.079 (0.142) | | 0.576 | -0.158 (0.162) | 0.330 |
| Age | -0.014 (0.011) | | 0.187 | 0.002 (0.010) | 0.870 |
| Time | 0.068 (0.074) | | 0.360 | -0.036 (0.058) | 0.538 |
| CU A+ vs. CU A- | 0.189 (0.194) | | 0.333 | -0.004 (0.189) | 0.985 |
| MCI A+ vs. CU A- | 0.224 (0.195) | | 0.253 | 0.245 (0.194) | 0.208 |
| Baseline Z(FTP SUVR) * Time | -0.112 (0.075) | | 0.140 | 0.080 (0.081) | 0.328 |
| Baseline Z(FTP SUVR) * CU A+ | -0.152 (0.174) | | 0.384 | 0.054 (0.191) | 0.776 |
| Baseline Z(FTP SUVR) * MCI A+ | -0.050 (0.150) | | 0.739 | 0.042 (0.164) | 0.800 |
| Time * Age | -0.010 (0.007) | | 0.124 | -0.006 (0.005) | 0.259 |
| Time * CU A+ | -0.074 (0.114) | | 0.519 | 0.065 (0.096) | 0.500 |
| Time * MCI A+ | -0.051 (0.115) | | 0.658 | -0.055 (0.094) | 0.599 |
| Baseline Z(FTP SUVR) * CU A+ * Time | 0.119 (0.097) | | 0.223 | -0.012 (0.099) | 0.907 |
| Baseline Z(FTP SUVR) * MCI A+ * Time | 0.117 (0.083) | | 0.161 | -0.009 (0.084) | 0.913 |
| Baseline Precentral Predicting Annual Change in Precentral | | | | | |
| Baseline Z(FTP SUVR) | 0.064 (0.129) | | 0.621 | -0.019 (0.146) | 0.899 |
| Age | -0.016 (0.010) | | 0.125 | 0.007 (0.009) | 0.442 |
| Time | 0.072 (0.070) | | 0.303 | -0.055 (0.056) | 0.326 |
| CU A+ vs. CU A- | 0.103 (0.187) | | 0.583 | -0.108 (0.164) | 0.511 |
| MCI A+ vs. CU A- | 0.326 (0.184) | | 0.078 ^†^ | 0.152 (0.164) | 0.355 |
| Baseline Z(FTP SUVR) * Time | -0.096 (0.066) | | 0.146 | 0.046 (0.074) | 0.538 |
| Baseline Z(FTP SUVR) * CU A+ | -0.121 (0.186) | | 0.515 | -0.076 (0.187) | 0.687 |
| Baseline Z(FTP SUVR) * MCI A+ | 0.116 (0.180) | | 0.518 | -0.037 (0.156) | 0.814 |
| Time * Age | 0.010 (0.006) | | 0.109 | -0.007 (0.005) | 0.162 |
| Time * CU A+ | -0.042 (0.109) | | 0.704 | 0.115 (0.094) | 0.226 |
| Time * MCI A+ | -0.077 (0.107) | | 0.477 | -0.013 (0.088) | 0.887 |
| Baseline Z(FTP SUVR) * CU A+ * Time | 0.086 (0.109) | | 0.430 | -0.007 (0.101) | 0.943 |
| Baseline Z(FTP SUVR) * MCI A+ * Time | 0.037 (0.118) | | 0.755 | -0.030 (0.085) | 0.722 |

**Supplementary Table 3. Relation between z-score normalized baseline flortaucipir SUVR (Z(FTP SUVR)) with partial volume correction and CSF phosphorylated tau (pTau-181).**

|  | | Ref. Region: Eroded Subcortical WM | | Ref. Region: Inf. Cerebellum | |
| --- | --- | --- | --- | --- | --- |
|  | *B* (SE) | | *p* | *B* (SE) | *p* |
| Baseline IT | **2.803 (0.741)** | | **<0.001 ***** | **3.119 (0.609)** | **<0.001 ***** |
| Baseline IT | **2.917 (0.444)** | | **<0.001 ***** | **1.746 (0.252)** | **<0.001 ***** |
| Baseline IP | **2.044 (0.527)** | | **<0.001 ***** | **0.928 (0.243)** | **<0.001 ***** |
| Baseline MFG | **2.162 (0.661)** | | **0.001 **** | **1.123 (0.334)** | **0.001 **** |
| Baseline Precentral | 1.999 (1.274) | | 0.119 | **2.555 (0.856)** | **0.003 **** |

**Supplementary Table 4. Longitudinal change in z-score normalized flortaucipir SUVR (Z(FTP SUVR)) with partial volume correction within each group.** Age is controlled for in each model.

|  | | Ref. Region: Eroded Subcortical WM | | Ref. Region: Inf. Cerebellar GM | |
| --- | --- | --- | --- | --- | --- |
|  | *B* (SE) | | *p* | *B* (SE) | *p* |
| Longitudinal Change in Z(FTP SUVR) vs. 0 | | | | | |
| Entorhinal | | | | | |
| CU A- | 0.076 (0.039) | | 0.051 ^†^ | 0.055 (0.046) | 0.230 |
| CU A+ | 0.067 (0.042) | | 0.107 | 0.057 (0.049) | 0.237 |
| MCI A+ | 0.006 (0.045) | | 0.892 | -0.061 (0.053) | 0.249 |
| Inferior Temporal | | | | | |
| CU A- | 0.070 (0.045) | | 0.120 | 0.050 (0.054) | 0.353 |
| CU A+ | **0.212 (0.048)** | | **<0.001 ***** | **0.280 (0.057)** | **<0.001 ***** |
| MCI A+ | **0.314 (0.052)** | | **<0.001 ***** | **0.347 (0.062)** | **<0.001 ***** |
| Inferior Parietal | | | | | |
| CU A- | 0.030 (0.040) | | 0.461 | 0.012 (0.061) | 0.841 |
| CU A+ | **0.126 (0.043)** | | **0.004 **** | **0.185 (0.065)** | **0.004 **** |
| MCI A+ | **0.215 (0.047)** | | **<0.001 ***** | **0.238 (0.071)** | **<0.001 ***** |
| Middle Frontal Gyrus | | | | | |
| CU A- | 0.035 (0.035) | | 0.323 | 0.014 (0.045) | 0.753 |
| CU A+ | 0.070 (0.038) | | 0.065 ^†^ | 0.080 (0.048) | 0.099 ^†^ |
| MCI A+ | **0.150 (0.041)** | | **<0.001 ***** | 0.100 (0.053) | 0.058 ^†^ |
| Precentral Gyrus | | | | | |
| CU A- | 0.042 (0.037) | | 0.255 | 0.016 (0.036) | 0.668 |
| CU A+ | 0.054 (0.039) | | 0.173 | 0.067 (0.039) | 0.084 ^†^ |
| MCI A+ | **0.132 (0.043)** | | **0.002 **** | **0.098 (0.042)** | **0.020 *** |

**Supplementary Table 5. Longitudinal change in z-score normalized flortaucipir SUVR (Z(FTP SUVR)) with partial volume correct with fixed effects of Age, Time, Group, Age x Time, and Group x Time.**

|  | | Ref. Region: Eroded Subcortical WM | | Ref. Region: Inf. Cerebellum | |
| --- | --- | --- | --- | --- | --- |
|  | *B* (SE) | | *p* | *B* (SE) | *p* |
| Entorhinal | | | | | |
| Age | **0.050 (0.018)** | | **0.006 **** | 0.008 (0.019) | 0.677 |
| Time | 0.076 (0.039) | | 0.052 ^†^ | 0.055 (0.046) | 0.231 |
| CU A+ vs. CU A- | 0.584 (0.319) | | 0.069 ^†^ | **0.738 (0.332)** | **0.028 *** |
| MCI A+ vs. CU A- | **2.296 (0.329)** | | **<0.001 ***** | **3.357 (0.343)** | **<0.001 ***** |
| Age * Time | 0.004 (0.003) | | 0.218 | -0.001 (0.004) | 0.838 |
| CU A+ * Time | -0.009 (0.058) | | 0.878 | 0.003 (0.068) | 0.966 |
| MCI A+ * Time | -0.070 (0.061) | | 0.250 | -0.116 (0.081) | 0.104 |
| Inferior Temporal | | | | | |
| Age | -0.000 (0.025) | | 0.986 | **-0.098 (0.040)** | **0.016 *** |
| Time | 0.070 (0.045) | | 0.121 | 0.050 (0.054) | 0.354 |
| CU A+ vs. CU A- | 0.769 (0.443) | | 0.084 ^†^ | 1.289 (0.712) | 0.072 ^†^ |
| MCI A+ vs. CU A- | **2.932 (0.458)** | | **<0.001 ***** | **5.646 (0.735)** | **<0.001 ***** |
| Age * Time | -0.001 (0.004) | | 0.852 | **-0.010 (0.005)** | **0.033 *** |
| CU A+ * Time | **0.142 (0.067)** | | **0.035 *** | **0.230 (0.080)** | **0.004 **** |
| MCI A+ * Time | **0.244 (0.070)** | | **<0.001 ***** | **0.297 (0.084)** | **<0.001 ***** |
| Interior Parietal | | | | | |
| Age | -0.041 (0.022) | | 0.061 ^†^ | **-0.175 (0.043)** | **<0.001 ***** |
| Time | 0.030 (0.040) | | 0.462 | 0.012 (0.061) | 0.841 |
| CU A+ vs. CU A- | 0.708 (0.386) | | 0.068 ^†^ | 1.433 (0.757) | 0.060 ^†^ |
| MCI A+ vs. CU A- | **2.551 (0.398)** | | **<0.001 ***** | **5.794 (0.781)** | **<0.001 ***** |
| Age * Time | 0.000 (0.004) | | 0.895 | -0.003 (0.005) | 0.527 |
| CU A+ * Time | 0.096 (0.060) | | 0.110 | 0.173 (0.090) | 0.057 ^†^ |
| MCI A+ * Time | **0.185 (0.063)** | | **0.004 **** | **0.226 (0.095)** | **0.018 *** |
| Rostral Middle Frontal Gyrus | | | | | |
| Age | **-0.037 (0.018)** | | **0.049 *** | **-0.145 (0.031)** | **<0.001 ***** |
| Time | 0.035 (0.035) | | 0.324 | 0.014 (0.045) | 0.753 |
| CU A+ vs. CU A- | 0.491 (0.327) | | 0.135 | 0.908 (0.546) | 0.098 ^†^ |
| MCI A+ vs. CU A- | **1.727 (0.337)** | | **<0.001 ***** | **3.714 (0.564)** | **<0.001 ***** |
| Age * Time | 0.002 (0.003) | | 0.548 | -0.001 (0.004) | 0.703 |
| CU A+ * Time | 0.035 (0.053) | | 0.508 | 0.065 (0.067) | 0.331 |
| MCI A+ * Time | **0.115 (0.055)** | | **0.038 *** | 0.085 (0.071) | 0.228 |
| Precentral Gyrus | | | | | |
| Age | **0.029 (0.013)** | | **0.020 *** | -0.023 (0.017) | 0.164 |
| Time | 0.042 (0.037) | | 0.256 | 0.016 (0.036) | 0.668 |
| CU A+ vs. CU A- | 0.101 (0.223) | | 0.649 | 0.209 (0.294) | 0.479 |
| MCI A+ vs. CU A- | 0.435 (0.230) | | 0.060 ^†^ | **1.358 (0.304)** | **<0.001 ***** |
| Age * Time | 0.003 (0.003) | | 0.301 | -0.001 (0.003) | 0.684 |
| CU A+ * Time | 0.012 (0.055) | | 0.832 | 0.051 (0.054) | 0.341 |
| MCI A+ * Time | 0.090 (0.057) | | 0.118 | 0.082 (0.056) | 0.146 |

**Supplementary Table 6. Effects of z-score normalized baseline flortaucipir SUVR (Z(FTP SUVR)) predicting longitudinal change in Z(FTP SUVR) with partial volume correction.** Age is controlled for in each model. Contrasts are shown to summarize the association between baseline Z(FTP SUVR) and change in Z(FTP SUVR) over time within each group (Baseline Z(FTP SUVR) x Time), and to determine whether the association between baseline and change in Z(FTP SUVR) varied across groups (Baseline Z(FTP SUVR) x Time x Group).

|  | | Ref. Region: Eroded Subcortical WM | | Ref. Region: Inf. Cerebellum | |
| --- | --- | --- | --- | --- | --- |
|  | *B* (SE) | | *p* | *B* (SE) | *p* |
| Baseline EC Predicting Annual Change in EC | | | | | |
| Baseline Z(FTP SUVR) * Time Within Each Group | | | | | |
| CU A- | -0.112 (0.066) | | 0.092 ^†^ | -0.056 (0.097) | 0.563 |
| CU A+ | **0.125 (0.058)** | | **0.030 *** | 0.079 (0.077) | 0.308 |
| MCI A+ | -0.067 (0.036) | | 0.060 ^†^ | -0.059 (0.040) | 0.141 |
| Baseline Z(FTP SUVR) * Time * Group Interactions | | | | | |
| CU A+ vs. CU A- | **0.237 (0.086)** | | **0.007 **** | 0.135 (0.123) | 0.277 |
| MCI A+ vs. CU A- | 0.045 (0.077) | | 0.562 | -0.003 (0.106) | 0.980 |
| Baseline IT Predicting Annual Change in IT | | | | | |
| Baseline Z(FTP SUVR) * Time Within Each Group | | | | | |
| CU A- | -0.142 (0.078) | | 0.069 ^†^ | 0.209 (0.129) | 0.104 |
| CU A+ | 0.102 (0.056) | | 0.068 ^†^ | 0.052 (0.056) | 0.356 |
| MCI A+ | 0.054 (0.051) | | 0.284 | 0.035 (0.035) | 0.330 |
| Baseline Z(FTP SUVR) * Time * Group Interactions | | | | | |
| CU A+ vs. CU A- | **0.244 (0.094)** | | **0.011 *** | -0.157 (0.141) | 0.266 |
| MCI A+ vs. CU A- | **0.196 (0.096)** | | **0.043 *** | -0.175 (0.134) | 0.195 |
| Baseline IP Predicting Annual Change in IP | | | | | |
| Baseline Z(FTP SUVR) * Time Within Each Group | | | | | |
| CU A- | -0.079 (0.073) | | 0.282 | 0.190 (0.116) | 0.102 |
| CU A+ | 0.112 (0.068) | | 0.100 | **0.131 (0.053)** | **0.014 *** |
| MCI A+ | -0.013 (0.071) | | 0.857 | -0.023 (0.039) | 0.545 |
| Baseline Z(FTP SUVR) * Time * Group Interactions | | | | | |
| CU A+ vs. CU A- | 0.191 (0.097) | | 0.053 ^†^ | -0.059 (0.127) | 0.645 |
| MCI A+ vs. CU A- | 0.066 (0.106) | | 0.535 | -0.213 (0.124) | 0.087 ^†^ |
| Baseline MFG Predicting Annual Change in MFG | | | | | |
| Baseline Z(FTP SUVR) * Time Within Each Group | | | | | |
| CU A- | -0.093 (0.063) | | 0.139 | 0.072 (0.073) | 0.323 |
| CU A+ | 0.061 (0.070) | | 0.383 | 0.103 (0.054) | 0.055 ^†^ |
| MCI A+ | 0.012 (0.041) | | 0.764 | **0.053 (0.022)** | **0.018 *** |
| Baseline Z(FTP SUVR) * Time * Group Interactions | | | | | |
| CU A+ vs. CU A- | 0.154 (0.092) | | 0.098 ^†^ | 0.031 (0.090) | 0.734 |
| MCI A+ vs. CU A- | 0.105 (0.077) | | 0.174 | -0.019 (0.077) | 0.800 |
| Baseline Precentral Predicting Annual Change in Precentral | | | | | |
| Baseline Z(FTP SUVR) * Time Within Each Group | | | | | |
| CU A- | -0.100 (0.063) | | 0.113 | 0.066 (0.076) | 0.381 |
| CU A+ | -0.013 (0.085) | | 0.879 | 0.078 (0.061) | 0.205 |
| MCI A+ | -0.039 (0.100) | | 0.700 | 0.022 (0.041) | 0.598 |
| Baseline Z(FTP SUVR) * Time * Group Interactions | | | | | |
| CU A+ vs. CU A- | 0.087 (0.103) | | 0.399 | 0.011 (0.097) | 0.909 |
| MCI A+ vs. CU A- | 0.061 (0.122) | | 0.616 | -0.045 (0.086) | 0.605 |

**Supplementary Table 7. Baseline z-score normalized flortaucipir SUVR (Z(FTP SUVR)) predicting longitudinal change in Z(FTP SUVR).**

|  | | Ref. Region: Eroded Subcortical WM | | Ref. Region: Inf.  Cerebellar GM | |
| --- | --- | --- | --- | --- | --- |
|  | *B* (SE) | | *p* | *B* (SE) | *p* |
| Baseline EC Predicting Annual Change in EC | | | | | |
| Baseline Z(FTP SUVR) | 0.166 (0.132) | | 0.211 | 0.056 (0.173) | 0.748 |
| Age | -0.004 (0.011) | | 0.726 | 0.007 (0.013) | 0.568 |
| Time | 0.027 (0.077) | | 0.729 | -0.025 (0.100) | 0.802 |
| CU A+ vs. CU A- | 0.022 (0.197) | | 0.912 | -0.078 (0.242) | 0.748 |
| MCI A+ vs. CU A- | 0.067 (0.221) | | 0.762 | -0.020 (0.294) | 0.945 |
| Baseline Z(FTP SUVR) * Time | -0.112 (0.066) | | 0.094 ^†^ | -0.056 (0.097) | 0.564 |
| Baseline Z(FTP SUVR) * CU A+ | **-0.367 (0.155)** | | **0.019 *** | -0.203 (0.205) | 0.322 |
| Baseline Z(FTP SUVR) * MCI A+ | -0.169 (0.145) | | 0.245 | -0.058 (0.186) | 0.755 |
| Time * Age | 0.007 (0.007) | | 0.289 | -0.004 (0.008) | 0.632 |
| Time * CU A+ | -0.013 (0.117) | | 0.914 | 0.060 (0.145) | 0.681 |
| Time * MCI A+ | 0.033 (0.130) | | 0.798 | 0.091 (0.167) | 0.586 |
| Baseline Z(FTP SUVR) * CU A+ * Time | **0.237 (0.086)** | | **0.007 **** | 0.135 (0.123) | 0.277 |
| Baseline Z(FTP SUVR) * MCI A+ * Time | 0.045 (0.077) | | 0.562 | -0.003 (0.106) | 0.980 |
| Baseline IT Predicting Annual Change in IT | | | | | |
| Baseline Z(FTP SUVR) | 0.045 (0.147) | | 0.760 | -0.287 (0.220) | 0.195 |
| Age | -0.013 (0.013) | | 0.307 | 0.020 (0.014) | 0.154 |
| Time | 0.084 (0.085) | | 0.323 | 0.031 (0.100) | 0.757 |
| CU A+ vs. CU A- | 0.152 (0.225) | | 0.500 | -0.197 (0.260) | 0.452 |
| MCI A+ vs. CU A- | 0.214 (0.245) | | 0.384 | 0.115 (0.287) | 0.689 |
| Baseline Z(FTP SUVR) * Time | -0.142 (0.078) | | 0.072 ^†^ | 0.209 (0.129) | 0.106 |
| Baseline Z(FTP SUVR) * CU A+ | -0.064 (0.170) | | 0.708 | 0.444 (0.237) | 0.063 ^†^ |
| Baseline Z(FTP SUVR) * MCI A+ | -0.101 (0.163) | | 0.534 | 0.247 (0.225) | 0.274 |
| Time * Age | 0.007 (0.008) | | 0.349 | **-0.020 (0.009)** | **0.018 *** |
| Time * CU A+ | 0.002 (0.133) | | 0.987 | 0.204 (0.153) | 0.185 |
| Time * MCI A+ | 0.100 (0.146) | | 0.494 | 0.212 (0.168) | 0.210 |
| Baseline Z(FTP SUVR) * CU A+ * Time | **0.244 (0.094)** | | **0.011 *** | -0.257 (0.141) | 0.266 |
| Baseline Z(FTP SUVR) * MCI A+ * Time | **0.196 (0.096)** | | **0.043 *** | -0.165 (0.134) | 0.195 |
| Baseline IP Predicting Annual Change in IP | | | | | |
| Baseline Z(FTP SUVR) | -0.029 (0.151) | | 0.846 | **-0.496 (0.231)** | **0.033 *** |
| Age | 0.007 (0.012) | | 0.526 | **0.037 (0.014)** | **0.008 **** |
| Time | 0.094 (0.075) | | 0.215 | 0.061 (0.081) | 0.456 |
| CU A+ vs. CU A- | 0.296 (0.202) | | 0.144 | 0.180 (0.252) | 0.474 |
| MCI A+ vs. CU A- | 0.199 (0.225) | | 0.378 | 0.251 (0.277) | 0.366 |
| Baseline Z(FTP SUVR) * Time | -0.079 (0.073) | | 0.285 | 0.190 (0.116) | 0.105 |
| Baseline Z(FTP SUVR) * CU A+ | -0.072 (0.179) | | 0.688 | **0.491 (0.247)** | **0.048 *** |
| Baseline Z(FTP SUVR) * MCI A+ | 0.014 (0.176) | | 0.938 | 0.460 (0.237) | 0.054 ^†^ |
| Time * Age | -0.004 (0.007) | | 0.593 | **-0.028 (0.007)** | **<0.001 ***** |
| Time * CU A+ | -0.089 (0.120) | | 0.460 | -0.100 (0.134) | 0.941 |
| Time * MCI A+ | 0.137 (0.140) | | 0.330 | **0.335 (0.151)** | **0.029 *** |
| Baseline Z(FTP SUVR) * CU A+ * Time | 0.192 (0.097) | | 0.053 ^†^ | -0.059 (0.127) | 0.645 |
| Baseline Z(FTP SUVR) * MCI A+ * Time | 0.066 (0.106) | | 0.535 | -0.213 (0.124) | 0.087 ^†^ |
| Baseline rMFG Predicting Annual Change in rMFG | | | | | |
| Baseline Z(FTP SUVR) | 0.048 (0.124) | | 0.702 | -0.073 (0.158) | 0.646 |
| Age | -0.003 (0.010) | | 0.748 | 0.003 (0.011) | 0.793 |
| Time | 0.073 (0.070) | | 0.300 | -0.011 (0.062) | 0.855 |
| CU A+ vs. CU A- | 0.187 (0.182) | | 0.305 | 0.040 (0.192) | 0.835 |
| MCI A+ vs. CU A- | 0.150 (0.190) | | 0.431 | 0.215 (0.203) | 0.291 |
| Baseline Z(FTP SUVR) * Time | -0.093 (0.063) | | 0.141 | 0.072 (0.073) | 0.326 |
| Baseline Z(FTP SUVR) * CU A+ | -0.207 (0.163) | | 0.206 | -0.072 (0.186) | 0.702 |
| Baseline Z(FTP SUVR) * MCI A+ | -0.022 (0.137) | | 0.873 | -0.029 (0.161) | 0.858 |
| Time * Age | 0.007 (0.006) | | 0.317 | -0.006 (0.005) | 0.277 |
| Time * CU A+ | -0.067 (0.109) | | 0.544 | 0.038 (0.099) | 0.700 |
| Time * MCI A+ | -0.011 (0.113) | | 0.920 | 0.042 (0.101) | 0.677 |
| Baseline Z(FTP SUVR) * CU A+ * Time | 0.154 (0.092) | | 0.098 ^†^ | 0.031 (0.090) | 0.734 |
| Baseline Z(FTP SUVR) * MCI A+ * Time | 0.105 (0.077) | | 0.174 | -0.019 (0.077) | 0.800 |
| Baseline Precentral Predicting Annual Change in Precentral | | | | | |
| Baseline Z(FTP SUVR) | 0.044 (0.125) | | 0.727 | -0.050 (0.150) | 0.738 |
| Age | -0.006 (0.010) | | 0.537 | 0.006 (0.009) | 0.491 |
| Time | 0.079 (0.071) | | 0.267 | -0.013 (0.060) | 0.834 |
| CU A+ vs. CU A- | 0.140 (0.184) | | 0.448 | -0.054 (0.169) | 0.750 |
| MCI A+ vs. CU A- | 0.177 (0.187) | | 0.344 | 0.116 (0.175) | 0.508 |
| Baseline Z(FTP SUVR) * Time | -0.100 (0.063) | | 0.117 | 0.066 (0.076) | 0.384 |
| Baseline Z(FTP SUVR) * CU A+ | -0.184 (0.171) | | 0.282 | -0.080 (0.184) | 0.666 |
| Baseline Z(FTP SUVR) * MCI A+ | 0.087 (0.182) | | 0.634 | 0.045 (0.161) | 0.783 |
| Time * Age | 0.010 (0.007) | | 0.124 | -0.004 (0.005) | 0.442 |
| Time * CU A+ | -0.060 (0.111) | | 0.588 | 0.063 (0.096) | 0.512 |
| Time * MCI A+ | -0.037 (0.114) | | 0.749 | 0.011 (0.098) | 0.909 |
| Baseline Z(FTP SUVR) * CU A+ * Time | 0.087 (0.103) | | 0.399 | 0.011 (0.097) | 0.909 |
| Baseline Z(FTP SUVR) * MCI A+ * Time | 0.061 (0.122) | | 0.616 | -0.045 (0.086) | 0.605 |

**Supplementary Table 8. Mean (standard deviation) flortaucipir SUVR values from the CU A- group that were used for z-score normalization.**

|  | Eroded WM | Inferior Cerebellum |
| --- | --- | --- |
| No Partial Volume Correction | | |
| Amygdala | 1.017 (0.062) | 1.189 (0.114) |
| Entorhinal | 0.959 (0.080) | 1.119 (0.101) |
| Parahippocampal | 0.941 (0.063) | 1.098 (0.078) |
| Fusiform | 1.012 (0.051) | 1.182 (0.084) |
| Inferior Temporal | 1.020 (0.060) | 1.191 (0.090) |
| Middle Temporal | 0.987 (0.068) | 1.152 (0.088) |
| Superior Temporal | 0.913 (0.048) | 1.068 (0.089) |
| Transverse Temporal | 0.870 (0.052) | 1.018 (0.104) |
| Banks STS | 1.013 (0.044) | 1.185 (0.106) |
| Temporal Pole | 0.945 (0.070) | 1.103 (0.091) |
| Rostral ACC | 0.915 (0.046) | 1.071 (0.111) |
| Caudal ACC | 0.903 (0.059) | 1.056 (0.103) |
| PCC | 0.935 (0.057) | 1.093 (0.095) |
| Isthmus Cingulate | 0.927 (0.045) | 1.084 (0.090) |
| Supramarginal | 0.918 (0.057) | 1.072 (0.093) |
| Inferior Parietal | 0.951 (0.080) | 1.110 (0.095) |
| Superior Parietal | 0.871 (0.084) | 1.015 (0.087) |
| Precuneus | 0.942 (0.057) | 1.100 (0.082) |
| Frontal Pole | 0.828 (0.081) | 0.966 (0.097) |
| Medial OFC | 0.944 (0.043) | 1.105 (0.109) |
| Lateral OFC | 1.013 (0.041) | 1.185 (0.107) |
| Pars Orbitalis | 0.983 (0.070) | 1.148 (0.106) |
| Pars Triangularis | 0.948 (0.055) | 1.108 (0.103) |
| Pars Opercularis | 0.925 (0.048) | 1.083 (0.107) |
| Rostral MFG | 0.894 (0.060) | 1.044 (0.089) |
| Caudal MFG | 0.881 (0.057) | 1.029 (0.094) |
| Superior Frontal | 0.852 (0.055) | 0.995 (0.088) |
| Insula | 0.946 (0.038) | 1.108 (0.109) |
| Cuneus | 0.932 (0.063) | 1.087 (0.082) |
| Pericalcarine | 0.950 (0.049) | 1.110 (0.089) |
| Lingual | 0.932 (0.054) | 1.087 (0.075) |
| Lateral Occipital | 0.935 (0.099) | 1.089 (0.093) |
| Paracentral | 0.878 (0.071) | 1.025 (0.090) |
| Precentral | 0.848 (0.051) | 0.991 (0.087) |
| Postcentral | 0.830 (0.065) | 0.968 (0.085) |
| Partial Volume Correction | | |
| Amygdala | 1.249 (0.179) | 1.036 (0.159) |
| Entorhinal | 1.849 (0.348) | 1.521 (0.229) |
| Parahippocampal | 1.537 (0.267) | 1.264 (0.152) |
| Fusiform | 1.632 (0.211) | 1.346 (0.131) |
| Inferior Temporal | 1.894 (0.250) | 1.561 (0.135) |
| Middle Temporal | 1.819 (0.282) | 1.494 (0.132) |
| Superior Temporal | 1.665 (0.211) | 1.373 (0.125) |
| Transverse Temporal | 1.360 (0.230) | 1.124 (0.169) |
| Banks STS | 1.525 (0.179) | 1.262 (0.142) |
| Temporal Pole | 1.839 (0.255) | 1.517 (0.164) |
| Rostral ACC | 1.444 (0.171) | 1.199 (0.170) |
| Caudal ACC | 1.570 (0.250) | 1.297 (0.183) |
| PCC | 1.516 (0.204) | 1.251 (0.137) |
| Isthmus Cingulate | 1.583 (0.219) | 1.305 (0.128) |
| Supramarginal | 1.698 (0.253) | 1.397 (0.129) |
| Inferior Parietal | 1.794 (0.325) | 1.470 (0.152) |
| Superior Parietal | 1.818 (0.333) | 1.490 (0.152) |
| Precuneus | 1.598 (0.197) | 1.318 (0.114) |
| Orbitofrontal | 1.889 (0.214) | 1.562 (0.167) |
| Frontal Pars | 1.869 (0.256) | 1.541 (0.156) |
| MFG | 1.767 (0.284) | 1.452 (0.149) |
| Superior Frontal | 1.631 (0.225) | 1.345 (0.136) |
| Insula | 1.359 (0.142) | 1.127 (0.134) |
| Cuneus | 1.782 (0.289) | 1.464 (0.153) |
| Pericalcarine | 1.509 (0.266) | 1.241 (0.161) |
| Lingual | 1.625 (0.259) | 1.335 (0.130) |
| Lateral Occipital | 1.927 (0.447) | 1.570 (0.214) |
| Paracentral | 1.676 (0.238) | 1.381 (0.135) |
| Precentral | 1.586 (0.254) | 1.304 (0.134) |
| Postcentral | 1.768 (0.327) | 1.450 (0.162) |
